# Spatiotemporal transferability of multi-temporal random forest model for mapping *Euryale ferox* Salisb. plantations using Sentinel-2 imagery

**DOI:** 10.3389/fpls.2026.1932872

**Published:** 2026-09-03

**Authors:** Keyao Zhang, Song Wang, Chao Chen, Xiaoqun He, Miaoting Cai, Xuewei Li, Jiang Long, Xiaoqing Wang

**Affiliations:** Jiangxi Quality Monitoring and Technology Service Center for Chinese Materia Medica Raw Materials, Jiangxi Research Center for Protection and Development of Traditional Chinese Medicine Resources, Jiangxi Provincial Institute of Traditional Chinese Medicine, Nanchang, China

**Keywords:** aquatic plants, *Euryale ferox* Salisb., feature optimization, independent validation, machine learning, remote sensing, spatiotemporal transferability index, vegetation index

## Abstract

*Euryale ferox* Salisb. is a widely cultivated aquatic plant of high edible and medicinal value, but lack of reliable production information hinders effective cultivation planning. Existing studies on *Euryale ferox* Salisb. mapping remain limited and often fail to fully exploit phenological information, resulting in suboptimal classification accuracy. More importantly, the spatiotemporal transferability of classification models, defined as the ability to maintain accuracy when applied to different regions or years without retraining, has been largely overlooked in crop mapping. To address this issue, we innovatively propose the Spatiotemporal Transferability Index (STI), comprising two novel metrics, STI_F1 and STI_Overall, to systematically quantify model performance retention after cross-regional and cross-year transfer. Using Sentinel-2 imagery acquired over Yugan County in 2024, we optimized features from spectral bands, vegetation indices, texture metrics, and DEM, and then constructed single-temporal (May, July, August, September, and October) and multi-temporal Random Forest (RF) models for *Euryale ferox* Salisb. planting area extraction. The spatiotemporal transferability of these models was then independently validated in Jinxian County in 2025, and assessed using the proposed STI metrics. The results indicated that Band 1, Band 12, the NDVWI, and DEM were consistently retained as discriminative features throughout the entire growth period. Among the single-temporal RF models, those for July, August, and September achieved high overall accuracies, all exceeding 97%, while the multi-temporal RF model reached 99.87%. In the spatiotemporal transferability validation, the multi-temporal RF model demonstrated the strongest transferability, achieving an STI_F1 of 94.02%, an STI_Overall of 88.86%, an overall accuracy of 90.13%, a Kappa coefficient of 0.8728, and an F1-score of 93.83%. The proposed framework integrates multi-temporal RF classification with an STI-based transferability evaluation, enabling accurate and efficient mapping of *Euryale ferox* Salisb. planting areas. The framework shows potential for extension to other crops and regions, supporting sustainable agricultural decisions.

## 1 Introduction

*Euryale ferox* Salisb. is a floating-leaved aquatic plant with both edible and medicinal value, with Jiangxi Province being one of its major production areas in China. In recent years, the growing demand for functional foods and natural medicines has driven the continuous expansion of *Euryale ferox* Salisb. cultivation, making accurate and timely monitoring of its planting areas increasingly critical for yield estimation, market regulation, and sustainable agricultural planning (Xue et al., 2026). Nevertheless, the unique aquatic habitat of *Euryale ferox* Salisb. poses substantial challenges for conventional field surveys, resulting in a notable lack of reliable, spatially explicit information on its planting distribution and thus hindering evidence-based decision-making.

Traditional crop information statistics primarily rely on ground surveys, yet these methods are costly, time-consuming, and susceptible to subjective influences (Zhang et al., 2026). Remote sensing technology provides an efficient solution to this challenge, and recent studies based on Sentinel-2 and Landsat imagery have made progress in estimating the coverage of floating-leaved and emergent aquatic vegetation (Luo et al., 2023; Qin et al., 2025). However, research specifically targeting *Euryale ferox* Salisb. remains scarce, and existing remote sensing applications have predominantly relied on single temporal imagery, which fails to capture the full phenological cycle of the target crop and is susceptible to spectral confusion with other aquatic vegetation.

Effective crop classification from remote sensing imagery depends critically on the construction of an informative and compact feature space. However, the spectral characteristics of aquatic vegetation differ fundamentally from those of terrestrial crops due to the strong absorption and scattering effects of the water column (Villa et al., 2025). Sentinel-2 multispectral imagery provides the necessary spectral richness to address these challenges (Xin et al., 2025); texture features can compensate for the limitations of spectral information alone (Liu et al., 2023a); and topographic variables further contribute to classification accuracy. However, integrating multiple feature types inevitably introduces redundancy and increases model complexity, making feature selection an essential preprocessing step. In this study, we employ the Random Forest (RF) classifier, which provides built-in permutation importance measures based on out-of-bag data to facilitate systematic feature optimization (Mahmood et al., 2025).

A fundamental challenge in remote sensing-based crop identification is the spectral confusion arising from the “same spectrum for different objects” and “different spectra for the same object” problems (Yu et al., 2014). In complex aquatic environments where *Euryale ferox* Salisb. coexists with other vegetation such as lotus, single-temporal images are particularly susceptible to these spectral ambiguities. The same crop may exhibit markedly different spectral characteristics at different growth stages, while different crop types may display nearly identical spectra during certain phenological phases (Raza et al., 2026). To overcome this limitation, we constructed single-temporal RF models for multiple months across the growing season, each tailored to the corresponding phenological stage. More importantly, a multi-temporal model that integrates images from multiple key growth stages can leverage spectral feature combinations from different phenological periods to enhance the separability between crop types (Shen et al., 2025). By integrating spectral features from multiple phenological stages as independent inputs, multi-temporal approaches effectively incorporate information on crop growth and senescence patterns that single-date imagery cannot provide, thus substantially reducing spectral confusion and improving classification reliability.

Despite the strong performance of machine learning-based crop classification models within their source domains, their transferability across different spatial and temporal contexts remains a critical yet underexplored issue (Rusňák et al., 2023). Spatiotemporal transferability refers to the ability of a model trained on data from one region and time period to maintain satisfactory accuracy when applied to another region or year without retraining (Wijesingha et al., 2024; Wang et al., 2025). However, differences in crop phenology, atmospheric conditions, and soil backgrounds between source and target domains can substantially degrade model transfer performance (Hoppe et al., 2024). For instance, models trained on Sentinel-2 imagery from the U.S. counties showed considerable accuracy decline when transferred to fragmented agricultural landscapes in Armenia (Tiwari et al., 2026). This performance degradation is particularly pronounced in regions with significant spatiotemporal heterogeneity, and if not adequately evaluated prior to transfer, it can severely limit the reliability of models in operational monitoring applications.

More critically, a systematic evaluation framework for spatiotemporal transferability is currently lacking. Most existing studies have qualitatively described transfer performance by comparing accuracies between source and target domains, without a unified quantitative metric to directly assess the degree of performance retention during model transfer (Wijesingha et al., 2024). This deficiency makes it difficult to objectively compare the transferability of different models and to provide clear applicability criteria for operational deployment. However, systematic evaluation of spatiotemporal transferability for crop classification models remains relatively limited, and the extent to which multi-temporal feature integration can enhance model portability remains an open question that warrants rigorous investigation.

**Figure 1:**
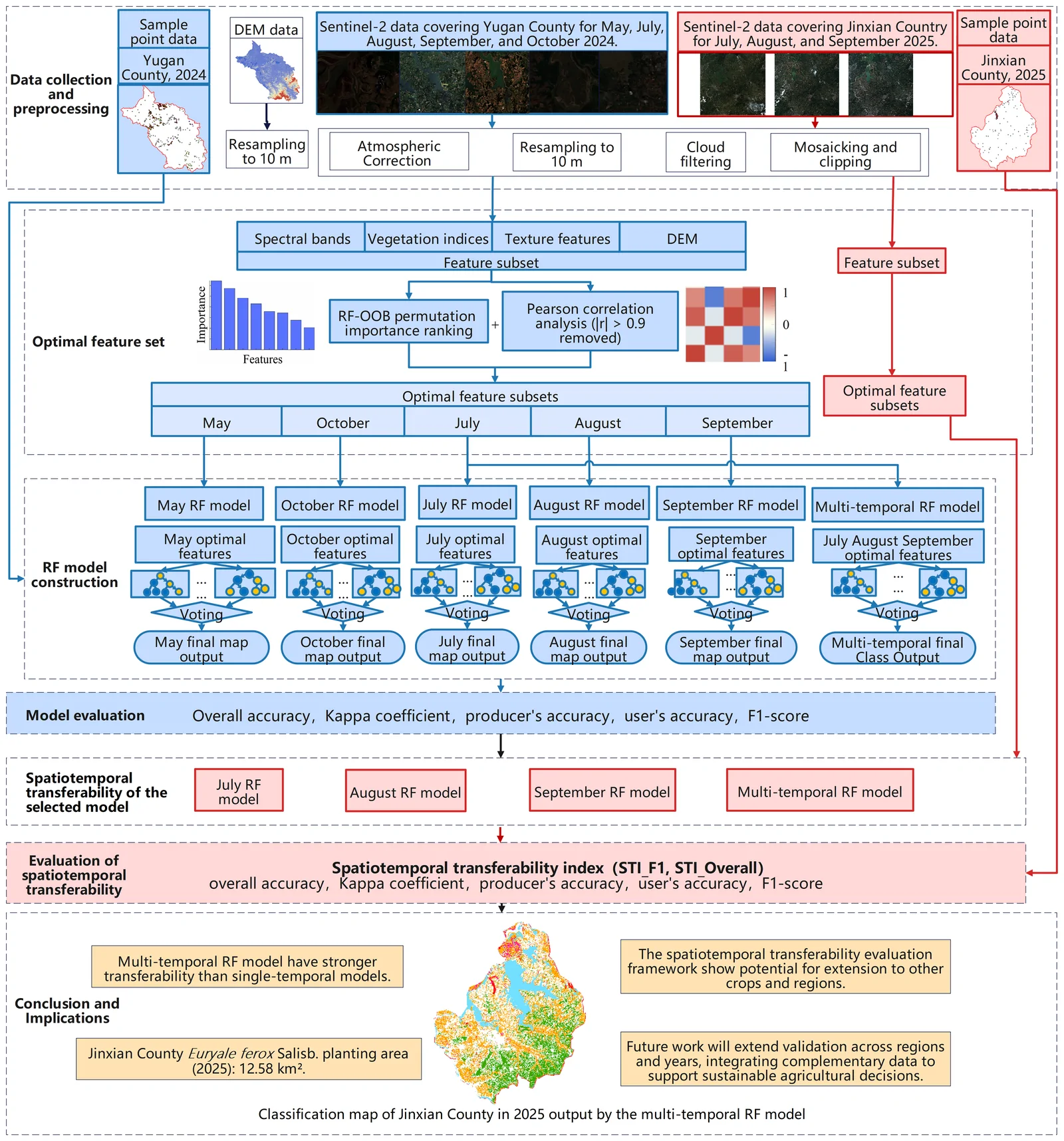
Flowchart of the methodology for *Euryale ferox* Salisb. planting area mapping. Different colors represent steps in the workflow: blue represents the workflow related to model construction; red represents the spatiotemporal transferability validation of the models; the white box represents the operations required for both model construction and validation; orange represents the main conclusions and implications.

To address the above mentioned challenges, this study innovatively proposes two evaluation metrics, STI_F1 and STI_Overall, which quantify the degree of performance retention for the target class (*Euryale ferox* Salisb.) and for all classes, respectively, after spatiotemporal transfer of the Random Forest models. These metrics provide a dynamic extrapolative perspective for systematically evaluating the cross-spatiotemporal transferability of both single-temporal and multi-temporal RF models. The specific objectives are: (1) to identify optimal feature subsets from a comprehensive pool of multi-source features across five key phenological months of *Euryale ferox* Salisb.; (2) to construct and compare five single-temporal RF models with an integrated multi-temporal RF model, quantifying the benefit of multi-temporal feature integration for addressing spectral confusion; and (3) to rigorously validate model transferability by applying the model trained in the source domain, Yugan County, 2024, to an independent target domain, Jinxian County, 2025, applying the proposed STI_F1 and STI_Overall metrics to quantify the degree of accuracy retention after transfer, and assessing conventional accuracy metrics while analyzing key factors influencing transferability. **Figure 1** summarizes the overall workflow of the proposed approach.

## 2 Materials and methods

### 2.1 Study area

**Figure 2:**
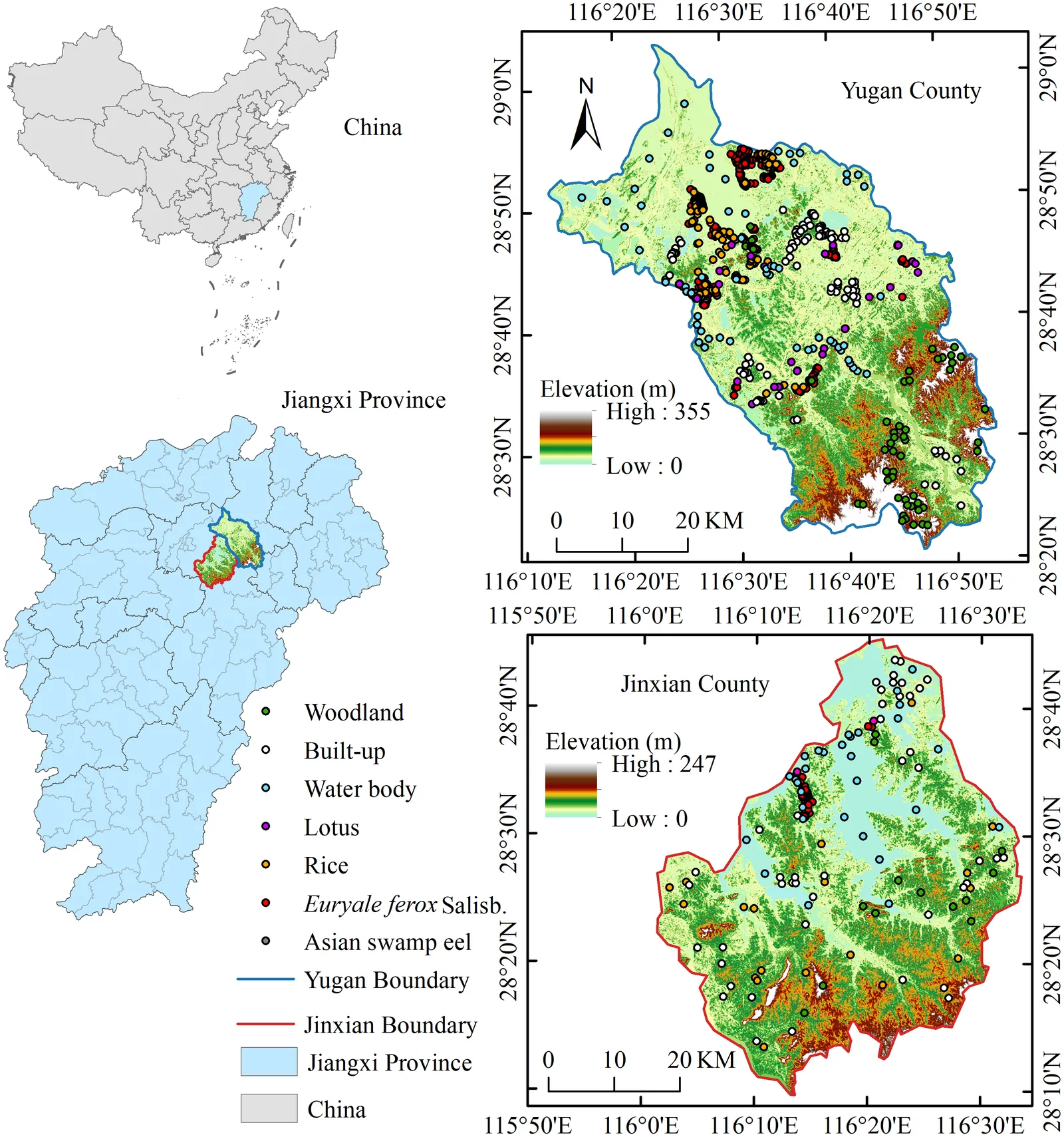
Geographic location of Yugan County and Jinxian County, along with the spatial distribution of sample points.

Jiangxi Province is one of the primary cultivation regions for *Euryale ferox* Salisb. in China. Within Jiangxi, Yugan County has the largest planting area, while Jinxian County ranks second. Accordingly, this study selected Yugan County as the source domain for model construction and Jinxian County as the independent target domain for validating model transferability. Both counties are situated in the southeastern Poyang Lake basin, with Yugan County spanning 28°21′–29°03′N, 116°13′–116°54′E and Jinxian County spanning 28°09′–28°46′N, 116°01′–116°33′E, as shown in **Figure 2**. The region has a subtropical monsoon climate with an average annual temperature of 17.8 °C and annual precipitation of 1400–1800 mm. The terrain is predominantly flat to gently undulating, with elevations ranging from 0 to 355 m above sea level. Soils are mainly derived from lacustrine and alluvial deposits, with high organic matter content and good water retention capacity, making them highly suitable for *Euryale ferox* Salisb. cultivation. *Euryale ferox* Salisb. cultivation is primarily concentrated in areas adjacent to Poyang Lake, where shallow water bodies with depths of 0.3–0.9 m provide optimal growing conditions.

### 2.2 Data

#### 2.2.1 Sample point data

Sample points were collected through a combination of field surveys and visual interpretation, with their quantities and locations presented in **Table 1**; **Figure 2**, respectively. The survey primarily collected information on land use types, crop species, growth cycles, *in situ* photographs, and the geographic coordinates of the sampling points. For land cover types that are difficult to identify directly from satellite imagery, namely *Euryale ferox* Salisb., Nelumbo nucifera Gaertn. (lotus), Oryza sativa L. (rice), and Asian swamp eel aquaculture ponds, all samples were acquired through field surveys, with GPS coordinates recorded at each location. For water bodies and woodland, a portion of samples was collected in the field while the remainder was obtained through visual interpretation of high-resolution imagery. For built-up areas, which are easily identifiable from imagery, all samples were generated solely through visual interpretation. Field sampling for model construction was conducted in Yugan County from October 15 to 18, 2023, during which crop type, growth stage, and surrounding environmental conditions were documented. Independent validation samples for spatiotemporal transferability assessment were collected in Jinxian County from October 22 to 24, 2025, following the same protocol. The model was constructed and validated at the pixel scale. Each sample point represented a land parcel, with the area and shape of the parcels determined by actual surface boundaries. Consistent with the 10 m × 10 m resolution of Sentinel-2 imagery, pixel values were extracted from all parcels, and each pixel was assigned a class label based on field surveys and visual interpretation. A total of 1,031,144 pixels, covering 103.11 km², were extracted from 567 sample parcels in Yugan County for model training and testing. In the spatiotemporal transferability test, 152 actual land cover type samples from Jinxian County were compared with the model outputs for independent validation.

**Table 1:** Information on modeling and independent validation samples.

Sample type	Sample size
*Euryale ferox* Salisb.	216
Rice	150
Lotus	35
Water body	91
Woodland	72
Built-up	138
Asian swamp eel	17
Total	719

#### 2.2.2 Acquisition and processing of remote sensing data

Sentinel-2 multispectral imagery was obtained from the European Space Agency’s Copernicus mission, which provides high-resolution optical data with 13 spectral bands spanning the visible, red-edge, near-infrared, and shortwave infrared regions (Ju et al., 2025). Level-2A bottom-of-atmosphere reflectance products were preferentially downloaded via the Copernicus Data Space Browser (https://browser.dataspace.copernicus.eu). For scenes where suitable Level-2A data were unavailable, Level-1C top-of-atmosphere reflectance products were acquired instead and subsequently processed to surface reflectance using the Sen2Cor processor. Twelve spectral bands (excluding band 10, the cirrus band) were utilized for analysis. A total of 10 scenes covering Yugan County from May to October 2024 were acquired for model construction, while 3 scenes covering Jinxian County from July to August 2025 were obtained for independent spatiotemporal validation. All scenes were selected with a cloud cover threshold of less than 25% to ensure favorable observation conditions during the key phenological stages of *Euryale ferox* Salisb.

Topographic information was derived from the ASTER Global Digital Elevation Model Version 003 (ASTER GDEM V003), which provides terrain data at a spatial resolution of 30 meters. DEM data were extracted and resampled to 10 m using bilinear interpolation to match the spatial resolution of the Sentinel-2 imagery, ensuring spatial consistency across all feature layers used in the classification.

All preprocessing procedures were implemented using Sen2Cor (versions 2.10 and 2.12), SNAP 11.0.0, ENVI 5.6, Matlab 2020b, and ArcMap 10.8. For scenes where only Level-1C data were available, atmospheric correction was performed using the Sen2Cor processor to convert top-of-atmosphere reflectance to Level-2A bottom-of-atmosphere surface reflectance. Subsequently, all 20 m and 60 m bands were resampled to a uniform 10 m spatial resolution via bilinear interpolation to align with the native 10 m bands. Cloud and cloud shadow pixels were identified and removed using the QA60 band to ensure that only clear-sky observations were retained for analysis. For each time period, multiple Sentinel-2 tiles were mosaicked to provide complete coverage of the study area, and the resulting composite images were clipped to the administrative boundaries of Yugan County and Jinxian County. Finally, labeled data points were created by combining field survey records and visual interpretation of high-resolution imagery, generating a comprehensive ground truth dataset to support model training and independent validation.

#### 2.2.3 Feature extraction

To capture key indicators for precision agriculture monitoring (such as vegetation vigor, water stress, topographic conditions), we extracted a total of 180 features from the preprocessed multi-temporal Sentinel-2 imagery. These features spanned four main categories: spectral bands, vegetation indices, texture features, and topographic information (represented by DEM). This comprehensive feature set was constructed to support the subsequent classification of *Euryale ferox* Salisb. planting areas.

For spectral band features, all 12 Sentinel-2 bands excluding Band 10 (B10, the cirrus band) were utilized, covering the visible, red-edge, near-infrared, and shortwave infrared spectral regions. Detailed information is provided in **Table 2**. These bands capture the fundamental reflectance characteristics of different land cover types and serve as the primary spectral inputs for the classification model.

**Table 2:** Sentinel-2 spectral bands used in this study.

Band name	Abbreviation	Description	Central wavelength (nm) (S2A | S2B)	Resolution (m)
Band 1	B1	Aerosol	442.7 | 442.2	60
Band 2	B2	Blue	492.4 | 492.1	10
Band 3	B3	Green	559.8 | 559.0	10
Band 4	B4	Red	664.6 | 664.9	10
Band 5	B5	Red Edge1	704.1 | 703.8	20
Band 6	B6	Red Edge2	740.5 | 739.1	20
Band 7	B7	Red Edge3	782.8 | 779.7	20
Band 8	B8	NIR	832.8 | 832.9	10
Band 8A	B8A	Red Edge4	864.7 | 864.0	20
Band 9	B9	Water Vapor	945.1 | 943.2	60
Band 11	B11	SWIR1	1613.7 | 1610.4	20
Band 12	B12	SWIR2	2202.4 | 2185.7	20

To enhance the discrimination between *Euryale ferox* Salisb. and other land cover types, a total of 15 vegetation indices were calculated from the Sentinel-2 bands. Detailed descriptions of the vegetation indices, texture features, and topographic features are shown in **Table 3**. These vegetation indices were selected to represent various biophysical and biochemical properties of vegetation canopies, including greenness, chlorophyll content, and water status. The inclusion of multiple vegetation indices provides complementary spectral information that can improve separability among crop types, particularly in aquatic environments where water background effects can alter single-band reflectance values.

**Table 3:** Details of vegetation indices, texture features, and topographic feature.

Feature categories	Features	Abbreviation	Formula	Reference
Vegetation features	Normalized Difference Vegetation Index	NDVI	$\text{NDVI}=\frac{\text{B}8-\text{B}4}{\text{B}8+\text{B}4}$	(Rouse et al., 1974)
	Enhanced Vegetation Index	EVI	$\text{EVI}=2.5\times \frac{\text{B}8-\text{B}4}{\text{B}8+6\times \text{B}4-7.5\times \text{B}2+1}$	(Rouse et al., 1974)
	Ratio Vegetation Index	RVI	$\text{RVI}=\frac{\text{B}8}{\text{B}4}$	(Priebe et al., 1999)
	Difference Vegetation Index	DVI	DVI=B8−B4	(Ibrahim et al., 2019)
	Renormalized Difference Vegetation Index	RDVI	$\text{RDVI}=\frac{\text{B}8-\text{B}4}{\sqrt{\text{B}8-\text{B}4}}$	(Roujean and Breon, 1995)
	Soil-Adjusted Vegetation Index	SAVI	$\text{SAVI}=\frac{\text{B}8-\text{B}4}{\text{B}8+\text{B}4+0.5}\times \left(1+0.5\right)$	(Huete, 1988)
	Green Normalized Difference Vegetation Index	GNDVI	$\text{GNDVI}=\frac{\text{B}8-\text{B}3}{\text{B}8+\text{B}3}$	(Julio Cezar Souza Vasconcelos et al., 2026)
	Normalized Difference Red Edge 1	NDRE1	$\text{NDRE}1=\frac{\text{B}6-\text{B}5}{\text{B}6+\text{B}5}$	(Clevers and Gitelson, 2013)
	Normalized Difference Chlorophyll Index	NDCI	$\text{NDCI}=\frac{\text{B}5-\text{B}4}{\text{B}5+\text{B}4}$	(Mohanty et al., 2024)
	Normalized Difference Vegetation Wetness Index	NDVWI	$\text{NDVWI}=\frac{\text{B}8-\text{B}11}{\text{B}8+\text{B}11}$	(Liu et al., 2017)
	Modified Normalized Difference Water Index	MNDWI	$\text{MNDWI}=\frac{\text{B}3-\text{B}11}{\text{B}3+\text{B}11}$	(Laghari et al., 2025)
	Greenness Index	GI	$\text{GI}=\frac{\text{B}3}{\text{B}4}$	(Zarco‐Tejada et al., 2005)
	Chlorophyll Concentration Reflectance Index	CCRI	$\text{CCRI}=\frac{\text{B}4}{\text{B}5}$	(Guo et al., 2025)
	Red-Green Ratio Index	RGRI	$\text{RGRI}=\frac{\text{B}4}{\text{B}3}$	(Fu et al., 2024)
	Transformed Vegetation Index	TVI	$\text{TVI}=\sqrt{\frac{\text{B}8-\text{B}4}{\text{B}8+\text{B}4}+0.5}$	(Deering et al., 1975)
Texture features	Grayscale Symbiosis Matrix	GLCM	Contrast Mean Variance Entropy Second Moment Homogeneity Dissimilarity Correlation	(Haralick et al., 1973)
Topographic feature	Digital Elevation Model	DEM	/	(Shukla et al., 2017)

For texture features, the GLCM method was employed to quantify the spatial heterogeneity of the imagery. However, extracting eight GLCM texture measures from each of the 12 spectral bands would result in 96 texture feature layers, introducing substantial redundancy and computational burden. To address this issue, Principal Component Analysis was first applied to the 12 Sentinel-2 bands. Subsequently, texture features including mean, variance, homogeneity, contrast, dissimilarity, entropy, second moment, and correlation were derived solely from the first principal component, which retained the largest proportion of spectral variance. This approach reduced the texture feature set to 8 meaningful layers each month while preserving the dominant spatial structure of the imagery.

Finally, DEM derived from the ASTER GDEM V003 dataset was included as a topographic feature. This variable accounts for terrain-driven variations in crop distribution, contributing to the overall classification accuracy. In total, the initial feature space consisted of 12 spectral bands, 15 vegetation indices, 8 texture features, and 1 topographic feature for each of the five months (May, July, August, September, and October), thereby providing 180 candidate features for subsequent feature selection.

### 2.3 Methods

#### 2.3.1 Feature optimization methods

Feature selection is a critical preprocessing step that aims to eliminate redundant and highly correlated variables, reduce model complexity, and enhance classification accuracy. In this study, a two-stage feature optimization procedure was implemented, combining Random Forest-based importance assessment with correlation analysis, and was performed exclusively on the training dataset. This procedure was independently applied to the feature sets of each phenological month (May, July, August, September, and October) to identify the optimal feature subsets tailored to the spectral characteristics of each growth stage.

In the first stage, the importance of each feature was evaluated using the RF built-in permutation importance measure based on out-of-bag (OOB) data. When training each RF model, approximately one-third of the samples are excluded from the bootstrap sample and constitute the OOB data. The OOB error is computed by predicting these held-out samples, and the importance of a given feature is quantified by measuring the increase in OOB error when the values of that feature are randomly permuted while all other features remain unchanged (Bladen and Cutler, 2024). A larger increase in OOB error indicates a more important feature for classification accuracy. For May, July, August, September, and October, all 36 initial features of each month were ranked in descending order of their importance scores.

In the second stage, pairwise Pearson correlation coefficients were calculated among all features to detect multicollinearity (Sheng and Zheng, 2023). The Pearson correlation coefficient measures the linear correlation between two features. When |r| > 0.9, two features are considered to have extremely strong correlation, indicating that they carry almost redundant information. A threshold of 0.9 has been widely adopted in machine learning feature selection practices (Ghiam et al., 2022; Khan and Alkhathami, 2024). In this study, highly correlated features with |r| > 0.9 were identified, and for each pair of highly correlated features, the one with the lower importance ranking was removed. This procedure ensures that retained features are both highly discriminative and minimally redundant, thereby improving model stability and interpretability. The two-stage optimization was performed separately for each of the five phenological months.

#### 2.3.2 Single-temporal and multi-temporal RF models

The RF algorithm was employed as the primary classifier for *Euryale ferox* Salisb. planting area extraction in this study. RF is an ensemble learning method that constructs a multitude of independent decision trees through bootstrap aggregation, with the final classification result determined by majority voting across all trees. For each tree, a bootstrap sample is drawn from the original training dataset, and at each node, a random subset of features is selected as the set of candidate features for splitting (Breiman, 2001). This dual randomization strategy significantly mitigates the risk of overfitting and enhances generalization capability compared to a single decision tree. RF is particularly well-suited for this study as it can effectively handle high-dimensional feature spaces with complex interactions among spectral bands, vegetation indices, texture measures, and topographic variables, while demonstrating strong robustness to noise and requiring no assumptions about the underlying data distribution.

Three key hyperparameters of the RF models were tuned using a grid search approach: the number of decision trees (NumTrees), the maximum tree depth (MaxDepth), and the minimum leaf node size (MinLeafSize). The feature sampling strategy at each split (MaxFeatures) was kept at its default value for classification (sqrt) (Probst et al., 2019). The grid search was conducted over a broad range of candidate values for each hyperparameter, and the optimal combination was selected based on the highest overall classification accuracy.

The growing season of *Euryale ferox* Salisb. spans from April to October. The seedling stage occurs from April to June, the flowering and fruiting stage from July to September, and the maturation and senescence stage in October. The complete growth cycle of *Euryale ferox* Salisb. spans from April to October. April was excluded because the plants had not yet emerged, resulting in limited spectral differences across the water surface. June was excluded due to the lack of usable Sentinel-2 imagery caused by persistent cloudy and rainy weather (Qu et al., 2026). Our study adopts a multi-temporal classification strategy, rather than a time series classification strategy. We used each available cloud-free image’s spectral features as independent input variables for the Random Forest classifier, without constructing any temporally dependent model. Therefore, the missing June image does not compromise the “integrity of input”, since there is no sequential dependency among the different time phases. Moreover, we are concerned that interpolation may introduce new errors.

Among the remaining months, we evaluated the single-temporal Random Forest models for May, July, August, September, and October. The results showed that July, August, and September yielded significantly higher classification accuracies than May and October, indicating that these three months capture the most critical phenological stages of *Euryale ferox* Salisb.—July represents the peak vegetative growth stage, August corresponds to the flowering and fruiting stage, and September covers the fruit ripening stage. Together, they provide a relatively complete phenological trajectory from peak growth to senescence. In addition, considering the relatively lower classification accuracies of May and October, we excluded them from the multi-temporal model to avoid introducing additional errors. Therefore, concatenating the features from July, August, and September to construct the multi-temporal model allows us to balance temporal coverage and classification accuracy (Wei et al., 2019). This multi-temporal approach effectively incorporates information on crop growth and senescence patterns that single-date imagery cannot provide, thereby substantially reducing the spectral confusion problems that frequently occur in aquatic environments where *Euryale ferox* Salisb. coexists with other aquatic vegetation.

All models were trained using the sample dataset collected in Yugan County in 2024. The sample dataset was randomly divided into training and validation subsets at a ratio of 7:3, with the training set used for model construction and hyperparameter tuning, and the validation set used for interim performance evaluation. In addition, 5-fold cross-validation was applied during model training to obtain a more robust estimate of model performance and to mitigate the risk of overfitting. In the 5-fold cross-validation procedure, the training set was further partitioned into five equal-sized folds; the model was iteratively trained on four folds and validated on the remaining fold, and the average accuracy across all five iterations was computed. This cross-validation strategy ensures that the reported model performance reflects genuine ability rather than artifacts of a particular data split. The classification performance of all single-temporal and multi-temporal RF models was systematically compared to quantify the benefit of multi-temporal feature integration. Finally, each model was applied to generate its corresponding classification map.

However, the initial classification results contained salt-and-pepper noise and small spurious patches due to misclassification. To enhance the spatial coherence of the planting areas and retain only meaningful planting patches, post-processing was performed on the binary mask extracted from the *Euryale ferox* Salisb. class. Morphological opening (disk-shaped structuring element, radius = 2 pixels) was first used to remove thin, elongated structures, followed by area-based filtering to eliminate connected components smaller than 10 pixels. The final classification map was then obtained.

#### 2.3.3 Accuracy assessment

The classification performance of all single-temporal and multi-temporal RF models was evaluated using confusion matrix-based metrics. The confusion matrix, also known as an error matrix, provides a comprehensive cross-tabulation of predicted versus actual land cover classes, serving as the foundation for deriving multiple quantitative accuracy indicators (Congalton, 1991). In this study, five evaluation metrics were calculated from the confusion matrix: producer’s accuracy (PA), user’s accuracy (UA), overall accuracy (OA), Kappa coefficient, and F1-score (Liu et al., 2023b).

PA, also referred to as recall, measures the proportion of reference pixels of a given class that are correctly identified, reflecting the classifier’s ability to avoid omission errors. UA, also termed precision, represents the proportion of pixels classified as a given class that actually belong to that class on the ground, indicating the classifier’s ability to avoid commission errors. OA is calculated as the ratio of correctly classified pixels to the total number of validation pixels, providing a general measure of classification performance across all classes. The Kappa coefficient measures the agreement between the classification results and the reference data, corrected for the expected agreement due to chance. The F1-score, defined as the harmonic mean of PA and UA, provides a balanced measure that considers both omission and commission errors, and is particularly useful for evaluating the classification performance of individual classes, such as *Euryale ferox* Salisb., especially when class distributions are imbalanced.

#### 2.3.4 Spatiotemporal transferability validation

A critical challenge in the operational application of remote sensing-based crop classification models is their transferability to new regions and time periods without requiring additional field surveys for model retraining. To evaluate the spatiotemporal transferability of the proposed multi-temporal RF model, an independent validation was conducted by directly transferring the model trained on 2024 data from Yugan County to 2025 data from Jinxian County, without any model retraining or parameter fine-tuning.

Jinxian County is located in the southeastern Poyang Lake basin, adjacent to Yugan County, and shares broadly similar climatic conditions and cropping systems. The validation dataset for Jinxian County was collected through field surveys conducted from October 22 to 24, 2025, supplemented by visual interpretation of high-resolution imagery. All samples from Jinxian County were used exclusively as independent test data. To validate model transferability, we fed the optimal feature subsets (extracted from July, August, and September 2025 Jinxian County Sentinel-2 imagery and DEM) into the single-temporal RF models (July, August, September) and the multi-temporal RF model. These models had all been constructed using the corresponding 2024 Yugan County features. No adjustments were made to the model structures, parameters, or feature weights. Specifically, the single-temporal RF models were used to evaluate spatiotemporal transferability when using single-date features, while the multi-temporal RF model was used to evaluate spatiotemporal transferability when using multi-temporal features.

To comprehensively assess transferability, PA, UA, OA, Kappa coefficient, and F1-score were calculated from the confusion matrix comparing the model-predicted classes and the ground-truth classes for Jinxian County in 2025. To quantify the model’s ability to retain classification performance after spatiotemporal transfer, we propose the Spatiotemporal Transferability Index (STI) as an evaluation framework. The STI comprises two complementary metrics: Spatiotemporal Transferability Index_F1 score (STI_F1), which focuses on the discriminative transferability for the target crop (*Euryale ferox* Salisb.), and Spatiotemporal Transferability Index_Overall (STI_Overall), which captures the overall transferability performance across all land cover classes. They are defined as:


$$STI\_F1=\frac{F1_{target}}{F1_{source}}$$

$$STI\_Overall=\frac{1}{2}\left(\frac{OA_{target}}{OA_{source}}+\frac{Kappa_{target}}{Kappa_{source}}\right)$$

where *F*1*_source_* and *F*1*_target_* denote the F1-scores for *Euryale ferox* Salisb. in the source domain (Yugan County, 2024) and target domain (Jinxian County, 2025), respectively; *OA_source_* and *Kappa_source_* are the overall accuracy and Kappa coefficient in the source domain; and *OA_target_* and *Kappa_target_* are the corresponding values in the target domain. For both STI_F1 and STI_Overall, values closer to 1 indicate better transferability, and a value of 1 signifies perfect transferability.

Distinction between STI and conventional accuracy metrics. Conventional accuracy metrics address a fundamental question: how well does a model perform within a specific spatiotemporal extent? They provide static, intrinsic evaluations of model performance in a given scenario. The STI, in contrast, addresses a different question: how much performance does the model retain when transferred to new spatiotemporal contexts? This is a dynamic, extrapolative evaluation of performance retention in a target domain. These two questions are scientifically distinct, yet the latter has received limited attention in remote sensing studies. Given that model accuracy often declines substantially after spatiotemporal transfer, the STI complements rather than replaces conventional metrics by providing an additional dimension for assessing model transferability.

To further investigate the factors influencing model transferability, the classification results between Jinxian County and Yugan County were compared, with particular attention paid to *Euryale ferox* Salisb. and those classes that exhibited significant declines in accuracy. Furthermore, by comparing the performance of single-temporal and multi-temporal RF models within Jinxian County, we quantified the extent to which multi-temporal feature integration enhances model portability in complex aquatic environments.

## 3 Results

### 3.1 Feature selection results

**Figure 3:**
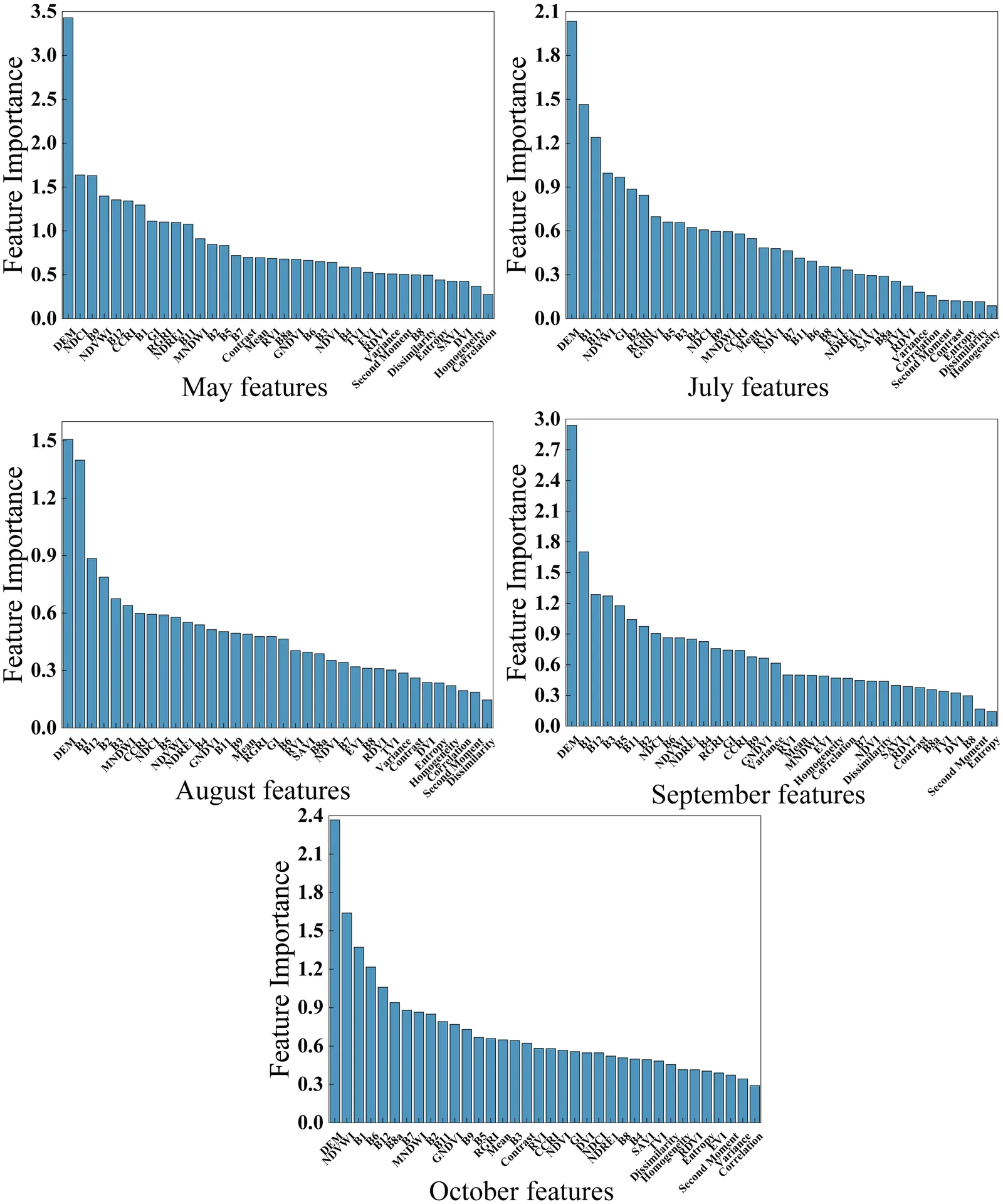
Feature importance ranking of the initial feature sets of monthly RF models. The abbreviations and full names of the features are listed below: DEM, Digital Elevation Model; B1 to B12 correspond to bands 1 through 12 of the Sentinel-2 satellite data. NDCI, Normalized Difference Chlorophyll Index; NDVWI, Normalized Difference Vegetation Wetness Index; CCRI, Chlorophyll Concentration Reflectance Index; GI, Greenness Index; RGRI, Red-Green Ratio Index; NDRE1, Normalized Difference Red Edge 1; MNDWI, Modified Normalized Difference Water Index; RVI, Ratio Vegetation Index; GNDVI, Green Normalized DifferenceVegetation Index; NDVI, Normalized DifferenceVegetation Index; TVI, Transformed Vegetation Index; EVI, Enhanced VegetationIndex; RDVI, Renormalized DifferenceVegetation Index; SAVI, Soil-Adjusted Vegetation Index; DVI, Difference Vegetation Index.

The two-stage feature optimization procedure combining the RF built-in permutation importance measure based on out-of-bag data with correlation analysis was applied to the initial set of 36 features for each of the five phenological months, and the importance rankings of the initial input feature sets for the five RF models are shown in **Figure 3**. In May, the top ten features by importance were DEM, NDCI, B9, NDVWI, B12, CCRI, B1, GI, RGRI, and NDRE1. In July, the top ten features were DEM, B1, B12, NDVWI, GI, B2, RGRI, GNDVI, B5, and B3. In August, the top ten features were DEM, B1, B12, B2, B3, MNDWI, CCRI, NDCI, B5, and NDVWI. In September, the top ten features were DEM, B1, B12, B3, B5, B11, B2, NDCI, B6, and NDVWI. In October, the top ten features were DEM, NDVWI, B1, B6, B12, B8A, B7, MNDWI, B2, and B11. Across all five months, DEM consistently ranked as the most important feature, followed by spectral bands and vegetation indices, while texture features generally ranked lower. Among the spectral bands, B1 and B12 were consistently among the top ten most important features in all five months, and among the vegetation indices, NDVWI was consistently among the top ten in all five months.

**Figure 4:**
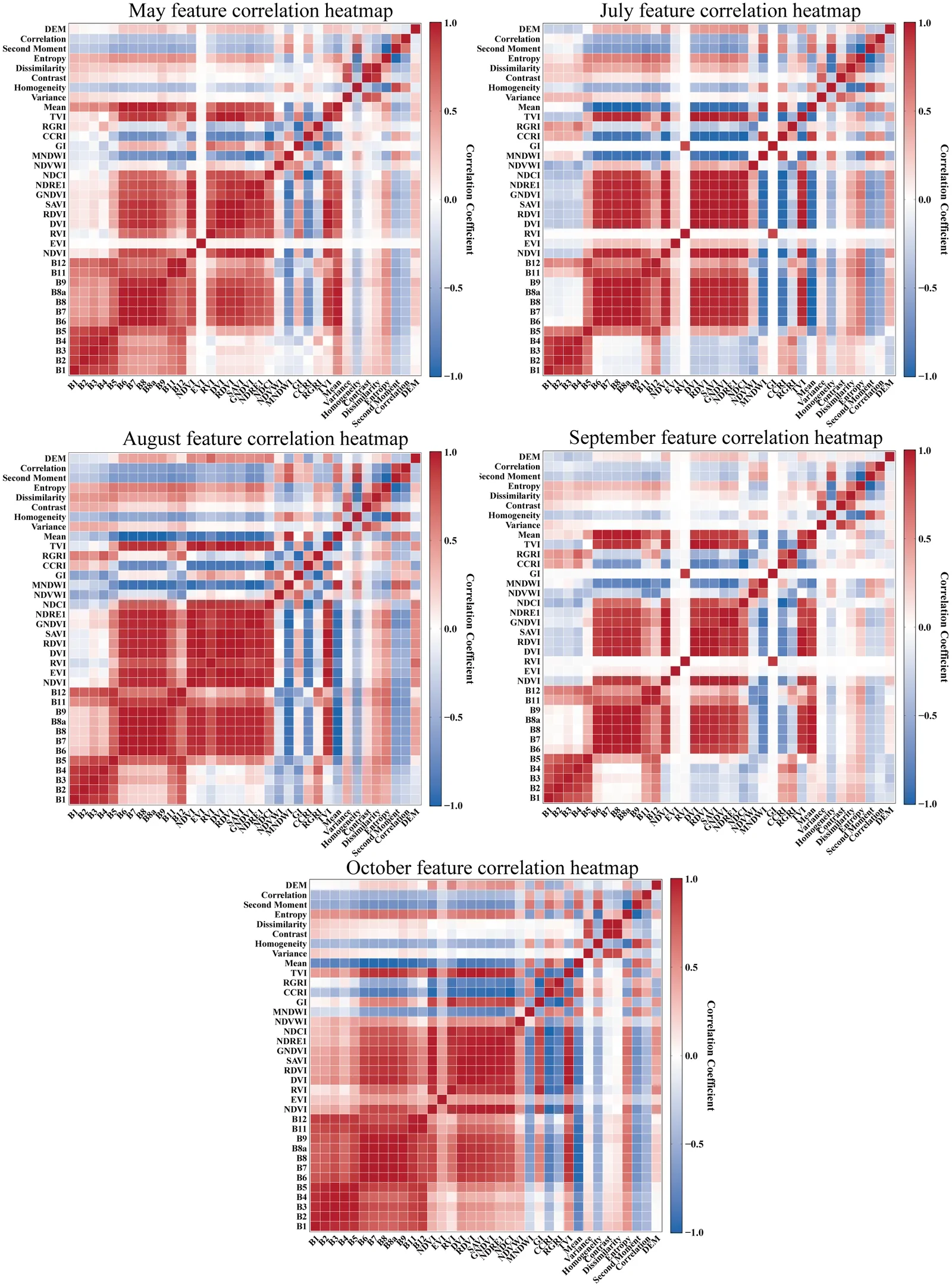
Feature correlation heatmaps of the initial feature sets of monthly RF models. The abbreviations and full names of the features are listed below: B1 to B12 correspond to bands 1 through 12 of the Sentinel-2 satellite data. NDVI, Normalized Difference Vegetation Index; EVI, Enhanced VegetationIndex; RVI, Ratio Vegetation Index; DVI, Difference Vegetation Index; RDVI, Renormalized DifferenceVegetation Index; SAVI, Soil-Adjusted Vegetation Index; GNDVI, Green Normalized DifferenceVegetation Index; NDRE1, Normalized Difference Red Edge 1; NDCI, Normalized Difference Chlorophyll Index; NDVWI, Normalized Difference Vegetation Wetness Index; MNDWI, Modified Normalized Difference Water Index; GI, Greenness Index; CCRI, Chlorophyll Concentration Reflectance Index; RGRI, Red-Green Ratio Index; TVI, Transformed Vegetation Index; DEM, Digital Elevation Model.

From the heatmap (**Figure 4**), several distinct correlation clusters are evident among the 36 features. Features with an absolute correlation coefficient greater than 0.9 were considered highly correlated, and of each such pair, the one with the higher importance ranking was retained. Across the five months, the DEM feature consistently exhibited low correlations with the other features. Strong correlations tended to occur among spectral bands; the same was observed for vegetation indices. In May, NDCI and CCRI exhibit a high negative correlation; B9 is highly positively correlated with B6, B7, B8, B8A, and Mean. In July, the absolute correlation coefficient between B1 and any other feature did not exceed 0.9, whereas B12 was highly correlated with B11. In August, B1 was highly correlated with B2 and B4, while B12 was highly correlated with B5 and B11. In September, B1 was highly correlated with B2, while B12 was highly correlated with B11. In October, the absolute correlation coefficient between NDVWI and any other feature did not exceed 0.9, whereas B1 was highly correlated with B2, B3, B4, and B5.

As shown in **Table 4**, the numbers of retained features were 10, 11, 12, 10, and 8 for May, July, August, September, and October, respectively. The variation in the size of the optimal feature subsets across months reflects the changing spectral separability between *Euryale ferox* Salisb. and other land cover types at different phenological stages. Despite the differences in feature subset sizes, several features were consistently retained across all five months, indicating their fundamental importance for *Euryale ferox* Salisb. discrimination throughout the entire growth period. B1 and B12 were among the consistently selected spectral bands, indicating that spectral information from both the visible and shortwave infrared regions contributes stably to *Euryale ferox* Salisb. discrimination across phenological stages. The NDVWI was also retained across all months, highlighting the critical role of water-related spectral indices in distinguishing floating-leaved aquatic vegetation from both terrestrial crops and open water. Furthermore, DEM was consistently selected as an important topographic feature, demonstrating that terrain-driven variations in cultivation suitability contribute to the spatial differentiation of *Euryale ferox* Salisb. planting areas. The texture features derived from the first principal component exhibited varying degrees of importance across months. In August, when vegetation cover reached its peak, variance, which reflects spatial heterogeneity, was preferentially retained.

**Table 4:** Optimized feature subsets selected for May, July, August, September, and October RF models.

May	July	August	September	October
DEM	DEM	DEM	DEM	DEM
B1	B1	B1	B1	B1
B2	B2	B3	B3	B6
B5	B5	B6	B4	B12
B9	B12	B12	B5	RGRI
B12	GI	CCRI	B6	GNDVI
GI	RGRI	MNDWI	B12	MNDWI
NDRE1	GNDVI	NDVWI	NDVWI	NDVWI
MNDWI	NDVWI	RVI	NDRE1	/
NDVWI	RVI	TVI	NDCI	/
NDCI	EVI	RGRI	/	/
/	/	Variance	/	/

Overall, the feature selection results confirm that multi-dimensional feature sets combining spectral, vegetation index, texture, and topographic information are essential for accurate *Euryale ferox* Salisb. classification, and that the optimal feature composition varies with phenological stage. The consistency of certain key features across all months also provides valuable guidance for simplifying future monitoring efforts by focusing on a core set of robust spectral and environmental variables.

### 3.2 Hyperparameter optimization results of the RF model

To achieve optimal classification performance, the hyperparameters of the RF models were tuned using a grid search approach. Three key hyperparameters were optimized: NumTrees, MaxDepth, and MinLeafSize. The MaxFeatures was kept at its default value for classification (sqrt). The initial preselected parameter ranges and the optimized results for each monthly RF model and the multi-temporal RF model are summarized in **Table 5**.

**Table 5:** Hyperparameter optimization results of single-temporal and multi-temporal RF models.

Hyperparameter	Initial preselected parameters	Optimized parameter results					
		May	July	August	September	October	Multi-Temporal
NumTrees	{50,100,125,130,135,140,142,143,144,145,146,148,150,160,175,200,250,350,500}	143	142	142	140	140	140
MaxDepth	{5,10,20,30,35,40,43,44,45,48,49,50,60,70}	49	49	49	45	45	45
MinLeafSize	{15,30,50,60,70,80,90,100,110,120,130,170,200,300,400,500,600,700,800,900}	600	100	100	130	130	130
Maxfeatures	sqrt	sqrt	sqrt	sqrt	sqrt	sqrt	sqrt

For the NumTrees parameter, a broad range of 19 candidate values from 50 to 500 was evaluated. The optimization results indicated that the optimal number of trees ranged from 140 to 143 across different models, with the May model requiring 143 trees, the July and August models each requiring 142 trees, and the September, October, and multi-temporal RF models converging at 140 trees. These relatively similar optimal values suggest that the ensemble size required for stable performance is largely consistent across phenological stages, and that approximately 140 trees are sufficient to achieve convergence without incurring unnecessary computational cost.

The MaxDepth parameter, controlling the maximum depth of each decision tree, was optimized over a range of 14 candidate values from 5 to 70. The optimal maximum depth was determined to be 49 for the May, July, and August models, and 45 for the September, October, and multi-temporal RF models. For the MinLeafSize parameter, which determines the minimum number of samples required at a leaf node, a wide range of 20 candidate values from 15 to 900 was tested. The May model required a substantially larger minimum leaf size of 600, suggesting that stronger regularization was necessary to prevent overfitting during the early growth stage when spectral signatures are less distinct. In contrast, the July and August models converged at a MinLeafSize of 100, while the September, October, and multi-temporal RF models converged at 130, reflecting a moderate level of regularization appropriate for the more stable spectral conditions during these periods. The MaxFeatures parameter was maintained at the default value of the square root of the total number of features for all models, a setting widely recommended for classification tasks (Louppe, 2014).

Overall, the grid search results demonstrate that while the optimal hyperparameters vary moderately across phenological stages, the performance of the RF model is relatively stable within a reasonable parameter range. The optimized parameters were subsequently used to train the final single-temporal and multi-temporal RF models for *Euryale ferox* Salisb. classification and spatiotemporal transferability validation.

### 3.3 Evaluation of single-temporal and multi-temporal RF models

The classification performance of the five single-temporal RF models and the multi-temporal RF model was systematically evaluated using an independent test set. The results are summarized in **Table 6**. The evaluation revealed clear performance differences among models, highlighting the critical influence of phenological stage selection on *Euryale ferox* Salisb. classification accuracy.

**Table 6:** Accuracy evaluation results of the RF model for the months of May, July, August, September, and October.

RF models	F1-score (%)	PA (%)	UA (%)	OA (%)	Kappa
May	89.94	84.88	95.64	95.13	0.9231
July	99.25	99.64	98.87	99.60	0.9936
August	99.46	99.45	99.46	99.52	0.9924
September	99.70	99.70	99.70	97.79	0.9650
October	59.61	46.18	84.05	83.49	0.7112
Multi-temporal	99.80	99.81	99.79	99.87	0.9979

F1, PA, and UA are for the *Euryale ferox* Salisb. class, while OA and Kappa are for all classes.

**Figure 5:**
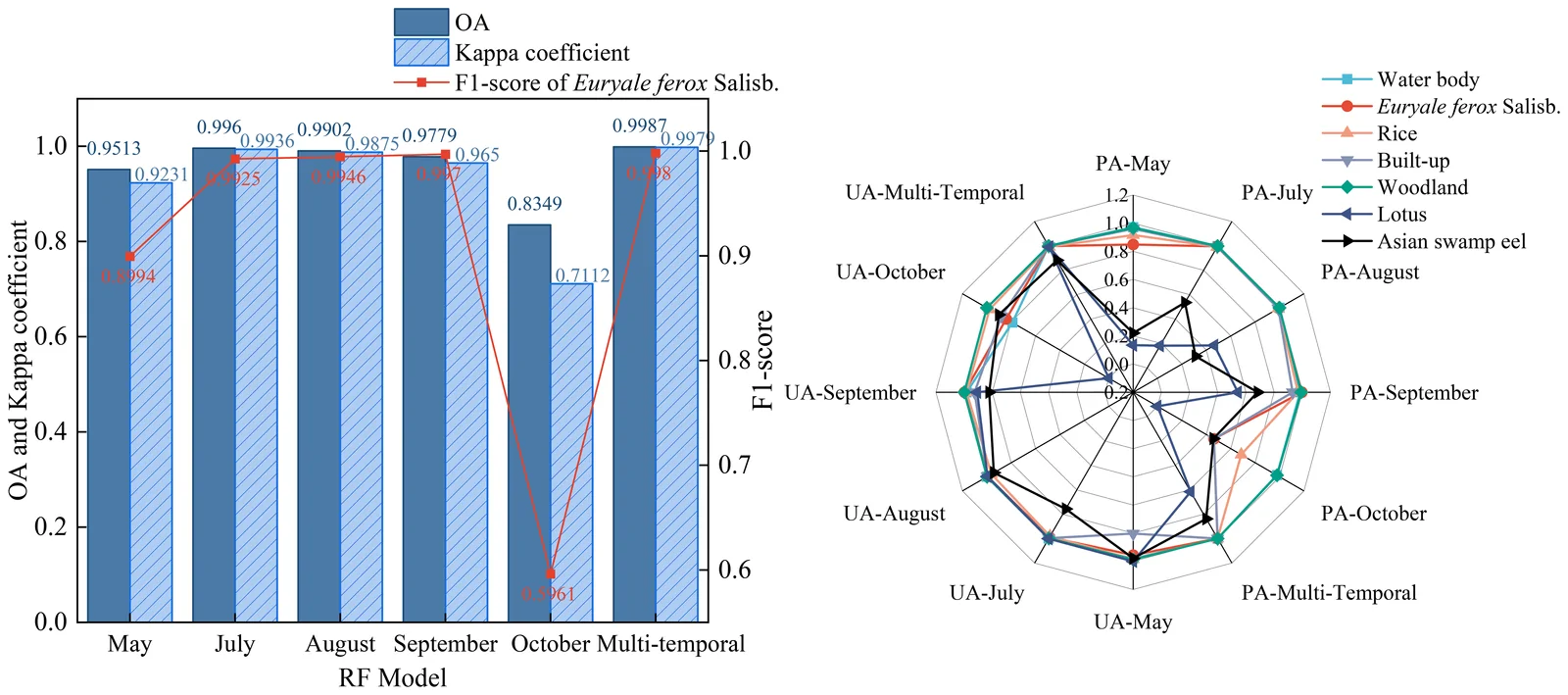
Accuracy evaluation plots for the May, July, August, September, October, and multi-temporal RF models. **(A)** OA and Kappa coefficient (overall metrics), and F1-score for *Euryale ferox* Salisb. across six RF models; **(B)** PA and UA for seven land cover classes across six RF models.

For single‑temporal models, in terms of overall classification performance across all seven land cover types (**Figure 5A**), the July RF model achieved the highest OA of 99.60% and a Kappa coefficient of 0.9936, followed by the August RF model with an OA of 99.52% and a Kappa coefficient of 0.9924. The July, August, and September RF models all attained OA exceeding 97%, forming a high-performance cluster during the peak growth period. The May RF model achieved an OA of 95.13%, surpassing 90%, while the October model exhibited considerably lower performance with an OA of 83.49% and a Kappa coefficient of 0.7112. This seasonal pattern reflects the progressive improvement in spectral separability as *Euryale ferox* Salisb. develops from the seedling stage toward full maturity, followed by a decline during senescence.

**Figure 5B** presents the PA and UA of all seven land cover classes across the single-temporal and multi-temporal RF models. Compared to the May and October models, the July, August, September, and multi-temporal RF models achieved higher classification accuracy for *Euryale ferox* Salisb. Specifically, the multi-temporal RF model achieved a PA of 99.81% and a UA of 99.79% for *Euryale ferox* Salisb. The September RF model also performed excellently, attaining a PA of 99.70% and a UA of 99.70%. Water body, rice, built-up and woodland maintained consistently high accuracies in the July, August, September, and multi-temporal RF models (PA > 96%, UA > 96%), reflecting their stable and distinctive spectral signatures. However, the accuracy of lotus and Asian swamp eel was substantially lower than that of the aforementioned classes.

The multi-temporal RF model, integrating features from July, August, and September, achieved the highest OA of 99.87% and a Kappa coefficient of 0.9979, outperforming all single-temporal RF models. For the *Euryale ferox* Salisb. class, the PA reached 99.81%, UA reached 99.79%, and the F1-score was 99.80%. In terms of OA, the multi-temporal RF model improved by 4.98%, 0.27%, 0.35%, 2.13%, and 19.62% compared to the May, July, August, September, and October single-temporal models, respectively. In terms of Kappa coefficient, the multi-temporal RF model improved by 8.10%, 0.43%, 0.55%, 3.41%, and 40.31% compared to the May, July, August, September, and October single-temporal models, respectively. In terms of F1-score for *Euryale ferox* Salisb., the multi-temporal RF model improved by 10.96%, 0.55%, 0.34%, 0.10%, and 67.42% compared to the May, July, August, September, and October single-temporal models, respectively. In terms of PA for *Euryale ferox* Salisb., the multi-temporal RF model improved by 17.59%, 0.17%, 0.36%, 0.11%, and 116.13% compared to the May, July, August, September, and October single-temporal models, respectively. In terms of UA for *Euryale ferox* Salisb., the multi-temporal RF model improved by 4.34%, 0.93%, 0.33%, 0.09%, and 18.73% compared to the May, July, August, September, and October single-temporal models, respectively. The multi-temporal model achieved consistent accuracy improvements over each of the five single-temporal models (May, July, August, September, and October) across all five evaluation metrics (OA, Kappa coefficient, F1-score, PA, and UA).

**Figure 6:**
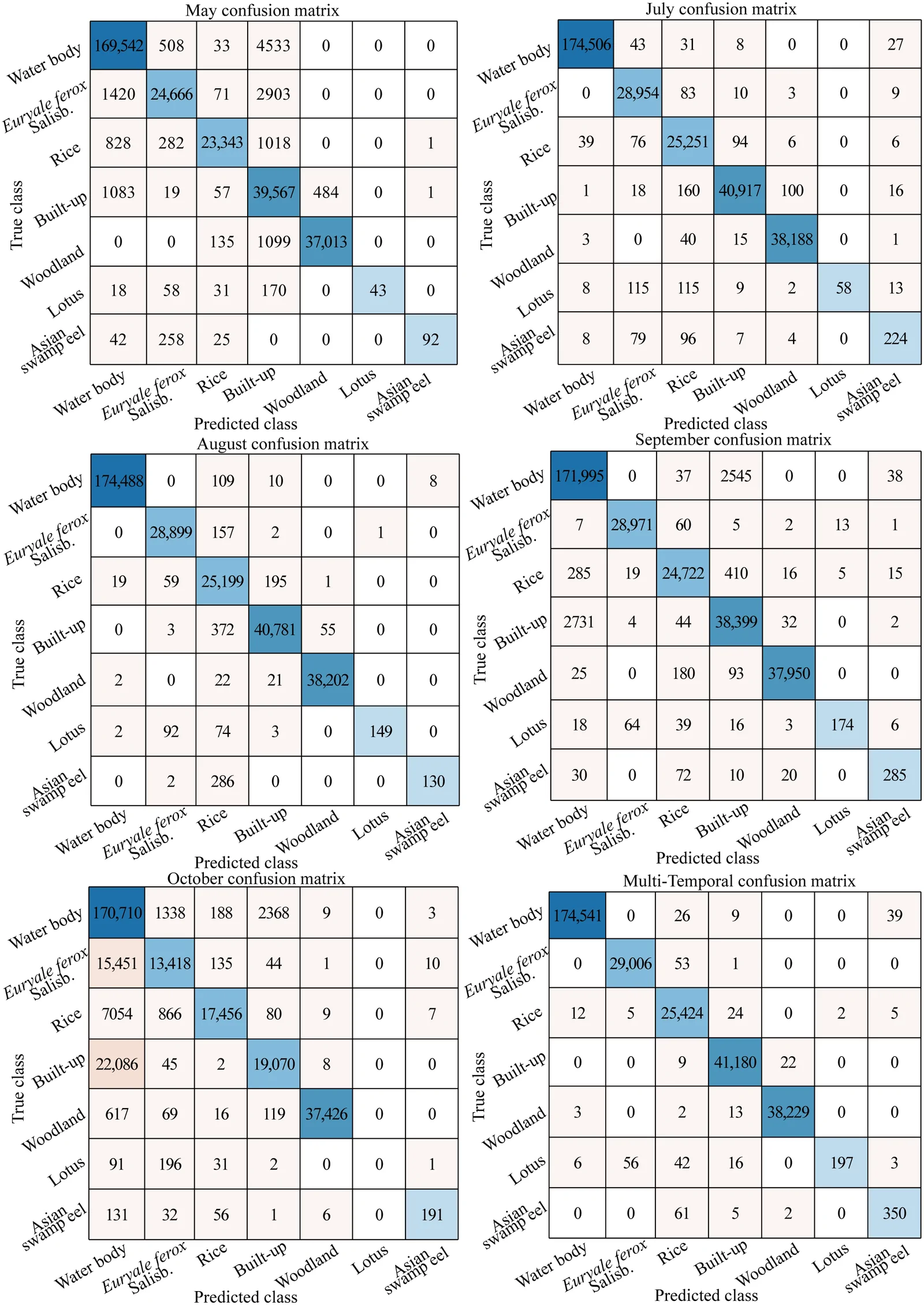
Confusion matrices for May, July, August, September, October, and the multi-temporal RF models. The blue diagonal cells indicate the number of correctly predicted pixels, where the predicted class matches the ground truth class. The off-white off-diagonal cells indicate the number of incorrectly predicted pixels, where the predicted class differs from the ground truth class.

The confusion matrices as shown in **Figure 6** provide detailed insight into the classification errors of each model. Based on the confusion matrices of the five single-month and multi-temporal RF models, in the May and October models, a relatively large number of *Euryale ferox* Salisb. pixels were misclassified as water bodies. For the remaining months, both omission and commission errors were low, with the number of correctly classified *Euryale ferox* Salisb. samples consistently exceeding 28,000. Notably, the multi-temporal RF model significantly reduced the number of *Euryale ferox* Salisb. pixels omitted or misclassified as water, rice, and lotus, achieving 29,006 correctly classified pixels, with only 115 omitted or misclassified pixels. The multi-temporal RF model substantially reduced these residual confusions by leveraging the complementary feature information available across different stages, resulting in highly accurate separation of all land cover classes. These results confirm that multi-temporal feature integration provides a robust solution to the spectral confusion problems commonly encountered in complex aquatic agricultural landscapes.

**Figure 7:**
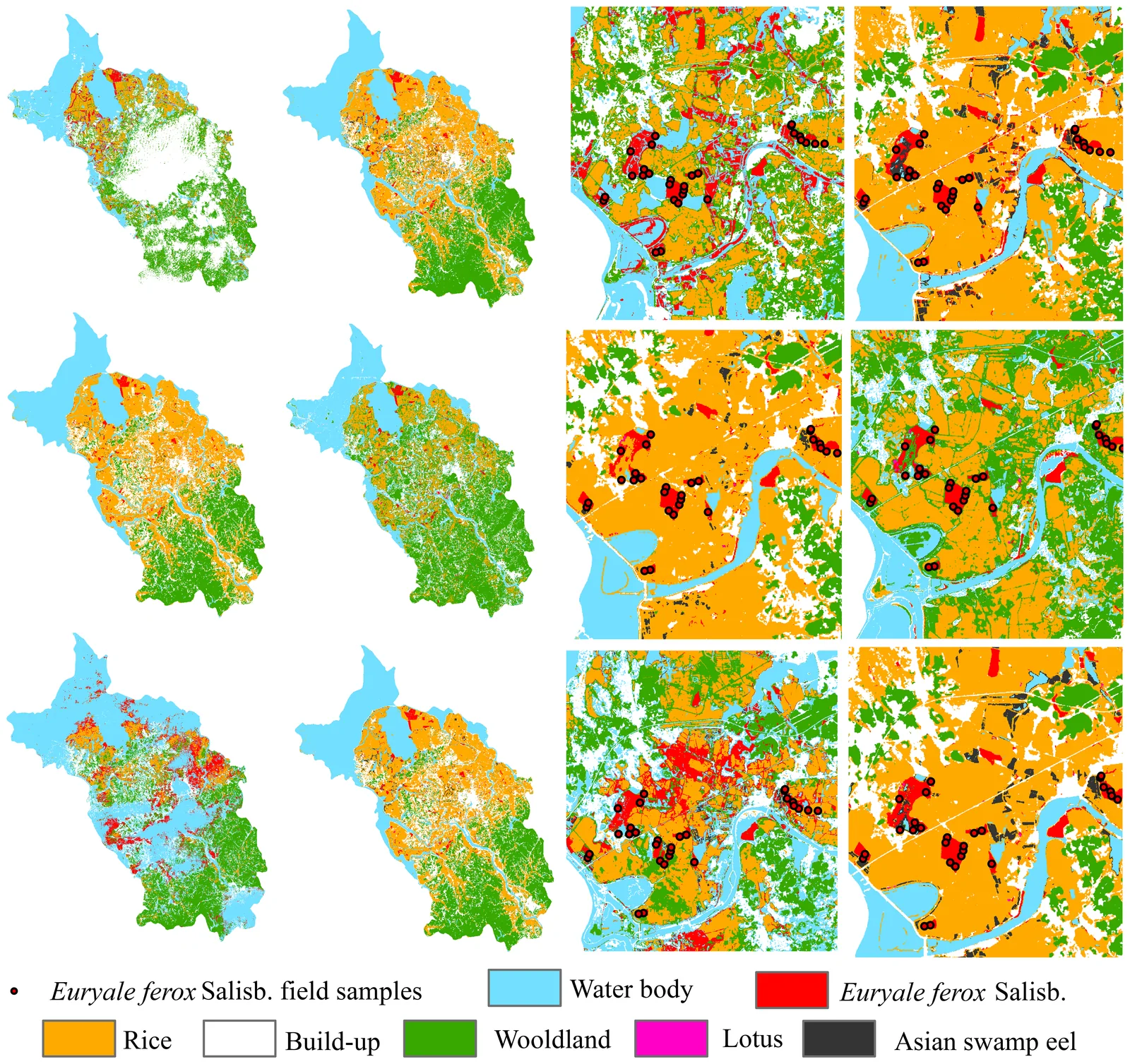
*Euryale ferox* Salisb. classification maps of Yugan County from the RF models for May **(A)**, July **(B)**, August **(C)**, September **(D)**, October **(E)**, and the multi-temporal **(F)**. **(A1–F1)** Local detailed views of **(A–F)**, respectively.

The classification maps of Yugan County output by the RF models for May, July, August, September, October, and the multi-temporal RF model in 2024 are shown in **Figure 7**. From the overall maps (A–F), it can be seen that the models achieved good classification results for water body, woodland, rice, and other land cover types. The detailed views (A1–F1) further reveal that for *Euryale ferox* Salisb., the predicted outputs of the July, August, September, and multi-temporal RF models are in good agreement with the ground truth, while misclassification is more evident in May and October.

### 3.4 Spatiotemporal transferability validation results

To evaluate the spatiotemporal transferability of the models, the trained models constructed using 2024 data from Yugan County were directly applied to the 2025 imagery of Jinxian County, using the corresponding optimized feature subsets as input, without any further training or parameter adjustment. **Table 7** shows the detailed evaluation results of the cross-spatiotemporal independent validation.

**Table 7:** Accuracy assessment of single-temporal and multi-temporal RF models for independent spatiotemporal validation (Jinxian County, 2025).

RF model	STI_F1 (%)	STI_Overal (%)	F1-score (%)	PA (%)	UA (%)	OA (%)	Kappa
July	88.46	87.43	87.80	85.71	90.00	88.67	0.8528
August	87.83	47.31	87.36	90.48	84.44	55.26	0.3879
September	90.27	74.96	90.00	85.71	94.74	76.32	0.6935
Multi-temporal	94.02	88.86	93.83	90.48	97.44	90.13	0.8728

The spatiotemporal transferability of the each model in the target domain (Jinxian County, 2025), as measured by STI_F1 and STI_Overall, is shown below. For the July, August, and September single-temporal RF models, the STI_F1 values for *Euryale ferox* Salisb. were 88.46%, 87.83%, and 90.27%, respectively., while the multi-temporal RF model achieved 94.02%, representing improvements of 6.29%, 7.05%, and 4.15% over the July, August, and September models, respectively. For all classes, when the July, August, and September single-temporal RF models were transferred to Jinxian County in 2025, the STI_Overall values were 87.43%, 47.31%, and 74.96%, respectively, while the multi-temporal RF model achieved 88.86%, representing improvements of 1.64%, 87.83%, and 18.54% over the July, August, and September models, respectively. The multi-temporal model achieved consistent STI improvements over each of the five single-temporal models, for both *Euryale ferox* Salisb. and overall model performance.

The spatiotemporal transferability of each model in the target domain (Jinxian County, 2025), as measured by conventional accuracy metrics, is shown below. The multi-temporal model outperformed all single-temporal models in both overall metrics (OA and Kappa) and *Euryale ferox* Salisb. metrics (F1-score and UA). In terms of OA, the multi-temporal RF model improved by 1.65%, 63.10%, and18.09% compared to the July, August, and September single-temporal models, respectively. In terms of Kappa coefficient, the multi-temporal RF model improved by 2.35%, 125.01%, and 25.85% compared to the July, August, and September single-temporal models, respectively. In terms of F1-score for *Euryale ferox* Salisb., the multi-temporal RF model improved by 6.87%, 7.41%, and 4.26% compared to the July, August, and September single-temporal models, respectively.

Among the three single-temporal RF models, the September model achieved the best transferability performance for the *Euryale ferox* Salisb. class, with an F1-score of 90.00%, a PA of 85.71%, and a UA of 94.74%. The July RF model followed with an F1-score of 87.80%, a PA of 85.71%, and a UA of 90.00%. The August RF model attained an F1-score of 87.36%, a PA of 90.48%, and a UA of 84.44%. In terms of overall classification performance, the July RF model achieved the highest OA of 88.67% and a Kappa coefficient of 0.8528, followed by the September RF model with an OA of 76.32% and a Kappa coefficient of 0.6935. The August RF model exhibited an OA of 55.26% and a Kappa coefficient of 0.3879, indicating that despite relatively strong performance for the *Euryale ferox* Salisb. class, substantial confusion persisted among the other classes.

**Figure 8:**
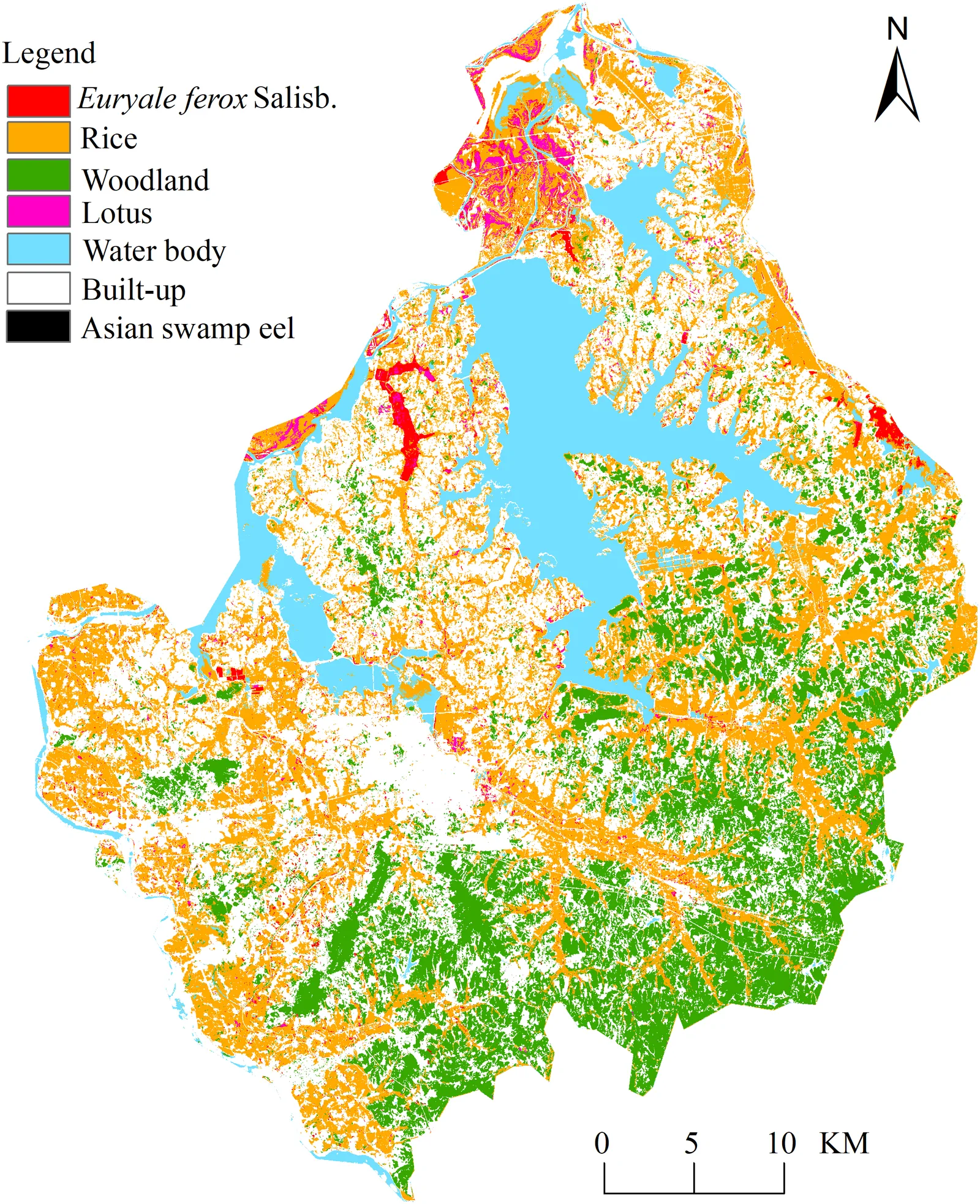
The classification map of Jinxian County in 2025 was generated based on the established multi-temporal RF model.

**Figure 8** shows the 2025 classification map of Jinxian County, which was output by the multi-temporal RF model constructed using 2024 Yugan County data, demonstrating the spatiotemporal transferability of the model. The model estimated the *Euryale ferox* Salisb. planting area to be 12.58 km², which is close to the field survey area of 11.67 km². There were 42 field sampling points for *Euryale ferox* Salisb. in Jinxian County in 2025. Of these, 38 were consistently identified as *Euryale ferox* Salisb. in the multi-temporal RF model-derived classification map, while 1 sampling point was classified as rice and 3 as lotus.

## 4 Discussion

### 4.1 Feature importance and phenological dynamics

The consistent retention of B1, B12, NDVWI, and DEM across all months reveals fundamental spectral characteristics governing aquatic crop discrimination. B1 (Coastal Aerosol, 443 nm) consistently maintained high importance as Sentinel-2’s only band specifically designed for atmospheric correction and water penetration. Its strong sensitivity to underwater information helps distinguish floating-leaved aquatic plants from water background by effectively capturing water-canopy interactions, while its sensitivity to aerosol scattering aids in removing atmospheric effects (Poursanidis et al., 2019). As water background conditions (turbidity, water depth) vary across different periods, B1 provides stable water-related discriminative information at each period. The persistent importance of B12 underscores a key distinction from terrestrial systems, where near-infrared and red bands typically dominate classification (Goldberg et al., 2021). B12 is highly sensitive to vegetation moisture content due to strong water absorption in the SWIR region (Rei et al., 2018). The moisture status of *Euryale ferox* Salisb. floating leaves varies across growth periods, with higher moisture and lower reflectance in early growth, and decreasing moisture with increasing reflectance as leaves age. This provides moisture-related discriminative information at each period, enabling effective differentiation of *Euryale ferox* Salisb. from lotus, rice, and other aquatic vegetation. In aquatic environments, the water background fundamentally alters canopy spectral response, making shortwave infrared bands particularly valuable for distinguishing floating-leaved plants from open water and emergent vegetation (Van Deventer et al., 2022). The consistent importance of NDVWI corroborates this interpretation. This index combines the sensitivity of vegetation indices to green vegetation with the sensitivity of water indices to water bodies, simultaneously capturing vegetation cover and water background information (Wang et al., 2012). For floating-leaved aquatic plants like *Euryale ferox* Salisb., the spectral signal is a mixture of canopy and water background: low cover with water-dominated background in early growth, peak cover with vegetation-dominated signal at mid-season, and canopy decline with renewed water exposure during senescence. NDVWI effectively utilizes the combined vegetation-water signal at each period to distinguish *Euryale ferox* Salisb. from other land cover types, which cannot be achieved by vegetation or water indices alone. DEM was also consistently selected, suggesting that even subtle topographic gradients influence cultivation suitability through indirect effects on water depth and drainage, thereby providing stable spatial constraints that complement temporally variable spectral features (Van Deventer et al., 2022).These findings suggest that future aquatic crop mapping should incorporate water-sensitive spectral features including SWIR bands, vegetation-water combined indices, and aerosol bands, rather than relying solely on terrestrial-oriented vegetation indices.

### 4.2 Effectiveness of multi-temporal feature integration for aquatic crop mapping

The multi-temporal Random Forest model outperformed all single-temporal models across multiple evaluation metrics in both the source and target domains. In particular, after cross-spatiotemporal transfer, the August single-temporal model exhibited a sharp decline in discriminative accuracy (55.26%) for overall classes, whereas the multi-temporal model maintained stable performance (OA = 90.13%). This can be primarily attributed to the following reason. The multi-temporal model treats the spectral features from July, August, and September as independent input variables. Different land cover types exhibit distinct combinations of features across these three periods. For example, the feature combination of rice in July, August, and September differs from that of *Euryale ferox* Salisb. and lotus. This distance disparity in the multi-dimensional feature space enables effective discrimination. Even if August features undergo drift, the independent feature dimensions from July and September still provide additional discriminative information, constraining samples within the correct class regions. This demonstrates the necessity of the multi-temporal strategy: the single-temporal model fails in transfer due to limited information, while the multi-temporal model significantly improves transfer robustness. A similar pattern has been reported in terrestrial crop classification studies (Wang et al., 2026).

OA and class-specific accuracy were not always consistent: July had the highest OA yet the lowest F1-score within the high-performance cluster. Likewise, in the cross-spatiotemporal validation, the August model yielded a low OA but a high F1-score for *Euryale ferox* Salisb., which is consistent with earlier findings (Masse et al., 2012). This underscores the importance of evaluating multiple metrics in model assessment.

### 4.3 Spatiotemporal transferability performance

The spatiotemporal validation results provide important insights into operational applicability. When transferred to Jinxian County in 2025, the multi-temporal RF model achieved an STI_F1 of 94.02% and an F1-score of 93.83% for *Euryale ferox* Salisb., an STI_Overall of 88.86%, an OA of 90.13%, and a Kappa coefficient of 0.8728 for all land cover classes, substantially outperforming all single-temporal RF models.

Several key factors affecting model transferability were identified through comparative analysis of the results from the independent validation area and the modeling area. First, phenological stage selection played a critical role. Among the single-temporal models, the July and September models exhibited relatively stable transferability for both *Euryale ferox* Salisb. and all classes (July: STI_F1 = 88.46%, STI_Overall = 87.43%; September: STI_F1 = 90.27%, STI_Overall = 74.96%). In contrast, while the August model maintained relatively stable transferability for *Euryale ferox* Salisb., its performance for all classes dropped markedly, with STI_Overall only 47.31%. This indicates that spectral characteristics at early and late peak growth stages are more stable across space and time than those at absolute peak canopy, when maximum canopy closure creates environmental context-dependent spectral signatures (Wolf et al., 2017). Second, land cover heterogeneity between the two counties. Confusion matrix analysis revealed that the August model severely over-predicted the built-up class, with water, forest, and rice predominantly misclassified into this category. Water misclassification can be attributed to regional differences in water optical properties: water bodies in Jinxian County in August, affected by algal blooms or high suspended sediment concentrations, exhibit elevated near-infrared reflectance (Wang, 2015), deviating from the source-domain relatively pure water template and approaching the spectral signature of built-up surfaces. In addition, the southern part of Jinxian County is a transitional zone from lakeshore plain to hills, where mixed pixels at 10 m resolution are inevitable, further pushing these pixels toward the built-up class. When land cover types in the target domain exhibit spectral characteristics that deviate from those learned in the source domain, the model tends to reassign them to spectrally similar classes with broader feature space coverage, ultimately degrading OA (Li et al., 2024). Third, inter-annual phenological shifts between different years. Despite both years being August, climatic conditions (temperature, precipitation, and Poyang Lake water levels) varied between 2024 and 2025, leading to inconsistent phenological states of crops at the same calendar date. The phenological-feature relationships learned from the August 2024 Yugan data underwent systematic shifts in the August 2025 Jinxian data, causing spectral features of certain land cover types to drift into the feature space of other classes from the source domain, further exacerbating misclassification.

### 4.4 Comparison with previous studies

Compared with previous remote sensing efforts targeting aquatic vegetation, the present study offers several methodological contributions. In this study, we proposed two novel evaluation metrics, STI_F1 and STI_Overall, to assess the spatiotemporal transferability of classification models, and evaluated the transferability of single-temporal and multi-temporal random forest models for *Euryale ferox* Salisb. classification. More recent studies have advanced aquatic vegetation mapping through improved algorithms and multi-source data integration. Early work by Wu et al. using multi-source satellite imagery and decision tree algorithms achieved 83% accuracy of *Euryale ferox* Salisb. extraction in Gaoyou Lake (Wu et al., 2017), demonstrating the feasibility of satellite-based aquatic crop monitoring, although their framework was relatively simple and limited to a single temporal approach without cross-spatiotemporal validation; a limitation that the present study seeks to fill. Luo et al. distinguished algal blooms, floating/emergent vegetation, and submerged vegetation at the vegetation type level by developing new vegetation indices combined with a decision tree algorithm, achieving an OA of 92.14% (Luo et al., 2023). Decision tree algorithms offer greater interpretability in the decision-making process. In contrast, the present study addresses species-level classification by employing a multi-temporal RF model, which inherently mitigates the overfitting issue common to single decision trees and better captures complex nonlinear relationships among spectral features through ensemble learning. Tufail et al. (2025) demonstrated the potential of deep learning models (CNNs and RNNs) for crop mapping using multi-temporal Sentinel-2 data and red-edge vegetation indices (Tufail et al., 2025). While these approaches offer powerful feature extraction capabilities, they typically require large training datasets and substantial computational resources. The RF model adopted in this study provides a pragmatic alternative with lower data and computational demands, while maintaining competitive accuracy. Xin et al. (2024) demonstrated the value of SAR-optical fusion for mapping submerged aquatic vegetation, achieving an OA of 88.89% (Xin et al., 2024). While such fusion enhances structural discrimination in complex aquatic environments, it also introduces additional data acquisition constraints and noise sensitivity. For species-level identification of floating-leaved aquatic crops, spectral features from multiple phenological stages, when combined as independent inputs, also can provide effective discriminative information. The multi-temporal RF classification framework and the associated STI evaluation system established in this study offer a methodological reference for species-level aquatic vegetation area mapping and independent cross-spatiotemporal validation. However, when applied to other floating-leaved aquatic plants, targeted parameter adjustments and feature optimization based on the phenology, spectral properties, and environmental context of the target species, along with independent accuracy validation, are required.

### 4.5 Limitations and future work

Several limitations of this study should be acknowledged. First, the transferability assessment is based on only one source region (Yugan County) and one target region (Jinxian County), and the datasets are limited to only two years (2024, 2025), which may not capture the full range of interannual variability in phenology and water conditions. Broader model applicability or large-scale operational implementation would require additional validation across more diverse regions, environmental conditions, and growing seasons. Future work could extend the study area and temporal scope to further test the model’s applicability under a wider range of conditions. Second, the current framework relies exclusively on Sentinel-2 optical imagery and DEM data, and the June image was missing due to persistent cloud cover. Although our multi-temporal strategy mitigates this issue to some extent by treating each available image as an independent input, the absence of a complete time series may still limit the model’s ability to capture full phenological information in cloud-prone regions. As discussed in Section 4.4, SAR-optical fusion introduces additional noise sensitivity. Nevertheless, in specific application scenarios where optical data are severely limited by persistent cloud cover, the systematic integration of SAR data into the multi-temporal analytical framework holds promise for filling temporal gaps in optical time series, and thus represents a worthwhile direction for future research. Third, this study employed only the Random Forest classifier; comparisons with other algorithms (e.g., deep learning models or support vector machines) were not conducted, and future work should explore whether these approaches could further improve transferability. Despite these limitations, the consistent performance of the multi-temporal model across source and target domains suggests that the proposed framework is robust for *Euryale ferox* Salisb. planting area extraction.

## 5 Conclusions

This study utilized Sentinel-2 imagery and optimized feature selection to construct a multi-temporal RF model for extracting *Euryale ferox* Salisb. planting areas, and systematically evaluated the model’s spatiotemporal transferability. The main conclusions are as follows:

1. Feature selection results demonstrated that B1, B12, the NDVWI, and DEM were retained across all phenological months, indicating their fundamental importance for aquatic crop discrimination throughout the entire growth period. The optimal feature subset sizes varied from 8 to 12 across months, reflecting changing spectral separability at different phenological stages.
2. In the source domain, the single-temporal RF models for July, August, and September achieved an OA exceeding 97%, forming a high-performance cluster during the peak growth period. The multi-temporal RF model attained the highest OA of 99.87% with a Kappa coefficient of 0.9979, confirming the value of integrating spectral information across multiple phenological stages.
3. In spatiotemporal transferability validation, the multi-temporal RF model achieved an OA of 90.13%, a Kappa coefficient of 0.8728, an STI_F1 of 94.02% for *Euryale ferox* Salisb., and an STI_Overall of 88.86% for all classes. For the single-temporal RF models of July, August, and September, the STI_Overall values were 87.43%, 47.31%, and 74.96%, respectively. The August model exhibited a marked decline in overall performance. In contrast, the multi-temporal RF model demonstrated substantially stronger spatiotemporal transferability.

These findings confirm that the proposed spatiotemporal transferability evaluation framework and the multi-temporal RF model for *Euryale ferox* Salisb. planting area extraction show potential for extension to other aquatic crops and regions. Future work could extend the validation to additional regions, incorporate multi-year data, and explore the integration of complementary data sources to further enhance model robustness and generalizability, thereby supporting decision-making, market regulation, and sustainable agricultural planning.

## Data Availability

The original contributions presented in the study are included in the article/**Supplementary Material**. Further inquiries can be directed to the corresponding author.

## References

[B1] Bladen K. Cutler D. R. (2024). Assessing agreement between permutation and dropout variable importance methods for regression and random forest models. Electron. Res. Arch. 32, 4495–4514. doi: 10.3934/era.2024203

[B2] Breiman L. J. M. L. (2001). Random forests. Mach. Learn. 45, 5–32. doi: 10.1023/A:1010933404324 41886696

[B3] Clevers J. G. P. W. Gitelson A. A. (2013). Remote estimation of crop and grass chlorophyll and nitrogen content using red-edge bands on Sentinel-2 and -3. Int. J. Appl. Earth Obs. Geoinf. 23, 344–351. doi: 10.1016/j.jag.2012.10.008 42574925

[B4] Congalton R. G. J. (1991). A review of assessing the accuracy of classifications of remotely sensed data. Remote Sens. Environ. 37, 35–46. doi: 10.1016/0034-4257(91)90048-B

[B5] Deering D. Rouse J. Haas R. Schell J. (1975). “ Measuring forage production of grazing units from Landsat MSS data”, in: Proceedings of 10th international symposium on remote sensing of environment (Ann Arbor, Michigan: ERIM).

[B6] Fu P. Li X. Zhang J. Ma C. Wang Y. Meng F. J. S. R. (2024). Remote sensing inversion on heavy metal content in salinized soil of Yellow River Delta based on Random Forest Regression—A case study of Gudao Town. Sci. Rep. 14, 11216. doi: 10.1038/s41598-024-62087-y 38755273 PMC11099045

[B7] Ghiam S. Eslahchi C. Shahpasand K. Habibi-Rezaei M. Gharaghani S. (2022). Identification of repurposed drugs targeting significant long non-coding RNAs in the cross-talk between diabetes mellitus and Alzheimer’s disease. Sci. Rep. 12, 18332. doi: 10.1038/s41598-022-22822-9 36316461 PMC9622874

[B8] Goldberg K. Herrmann I. Hochberg U. Rozenstein O. (2021). Generating up-to-date crop maps optimized for Sentinel-2 imagery in Israel. Remote Sens. 13, 3488. doi: 10.3390/rs13173488 30654563

[B9] Guo J. Huang X. Zhou D. Zhang J. Bao G. Tong S. . (2025). Estimation of the water content of needles under stress by Erannis jacobsoni Djak. via Sentinel-2 satellite remote sensing. Front. Plant Sci. 16, 1540604. doi: 10.3389/fpls.2025.1540604 40303864 PMC12038450

[B10] Haralick R. M. Shanmugam K. Dinstein I. H. J. I. T. O. S. man (1973). Textural features for image classification. IEEE J-STARS SMC-3, 610–621. doi: 10.1109/TSMC.1973.4309314 25079929

[B11] Hoppe H. Dietrich P. Marzahn P. Weiß T. Nitzsche C. Freiherr von Lukas U. . (2024). Transferability of machine learning models for crop classification in remote sensing imagery using a new test methodology: A study on phenological, temporal, and spatial influences. Remote Sens. 16, 1493. doi: 10.3390/rs16091493 30654563

[B12] Huete A. R. (1988). A soil-adjusted vegetation index (SAVI). Remote Sens. Environ. 25, 295–309. doi: 10.1016/0034-4257(88)90106-X

[B13] Ibrahim M. Ghanem F. Al-Salameen A. Al-Fawwaz A. M. J. I. J. O. G. (2019). The estimation of soil organic matter variation in arid and semi-arid lands using remote sensing data. Int. J. Geosci. 10, 576–588. doi: 10.4236/ijg.2019.105033

[B14] Ju J. Zhou Q. Freitag B. Roy D. P. Zhang H. K. Sridhar M. . (2025). The harmonized Landsat and Sentinel-2 version 2.0 surface reflectance dataset. Remote Sens. Environ. 324, 114723. doi: 10.1016/j.rse.2025.114723 42574925

[B15] Julio Cezar Souza Vasconcelos C. S. A. Eduardo Antonio Speranza João Francisco Gonçalves Antunes Luiz Antonio Falaguasta Barbosa Geraldo Magela de Almeida Cançado (2026). Estimating sugar yield in sugarcane using green normalized difference vegetation index derived from imagery obtained by remotely piloted aircrafts. Sugar Tech. 28, 667–674. doi: 10.1007/s12355-026-01737-z 30311153

[B16] Khan M. M. Alkhathami M. (2024). Anomaly detection in IoT-based healthcare: Machine learning for enhanced security. Sci. Rep. 14, 5872. doi: 10.1038/s41598-024-56126-x 38467709 PMC10928137

[B17] Laghari Y. Niu Z. Leghari S. J. Ali M. A. Li Q. Shi J. (2025). Wetland dynamics in the Indus River Delta: A Sentinel-2 and machine learning approach. J. Environ. Manage. 392, 126819. doi: 10.1016/j.jenvman.2025.126819 40782751

[B18] Li Z. Lu F. Zou J. Hu L. Zhang H. (2024). Generalized few-shot meets remote sensing: Discovering novel classes in land cover mapping via hybrid semantic segmentation framework. 2024 IEEE/CVF Conf. Comput. Vision Pattern Recognition Workshops (CVPRW). Seattle. 2744–2754. doi: 10.1109/cvprw63382.2024.00280 25079929

[B19] Liu Y. Song W. Deng X. (2017). Spatiotemporal patterns of crop irrigation water requirements in the Heihe River Basin, China. Water 9, 616. doi: 10.3390/w9080616 30654563

[B20] Liu J. Zhu Y. Song L. Su X. Li J. Zheng J. . (2023a). Optimizing window size and directional parameters of GLCM texture features for estimating rice AGB based on UAVs multispectral imagery. Front. Plant Sci. 14, 1284235. doi: 10.3389/fpls.2023.1284235 38192693 PMC10773816

[B21] Liu X. Xie S. Yang J. Sun L. Liu L. Zhang Q. . (2023b). Comparisons between temporal statistical metrics, time series stacks and phenological features derived from NASA Harmonized Landsat Sentinel-2 data for crop type mapping. Comput. Electron. Agric. 211, 108015. doi: 10.1016/j.compag.2023.108015 42574925

[B22] Louppe G. (2014). Understanding Random Forests: From Theory to Practice. Doctoral dissertation (Liège, Belgium: Universite de Liege, Belgium). doi: 10.13140/2.1.1570.5928

[B23] Luo J. Ni G. Zhang Y. Wang K. Shen M. Cao Z. . (2023). A new technique for quantifying algal bloom, floating/emergent and submerged vegetation in eutrophic shallow lakes using Landsat imagery. Remote Sens. Environ. 287, 113480. doi: 10.1016/j.rse.2023.113480 42574925

[B24] Mahmood T. Usman M. Conrad C. (2025). Selecting relevant features for random forest-based crop type classifications by spatial assessments of backward feature reduction. PFG- J. Photogramm. Remote Sens. Geoinf. Sci. 93, 173–196. doi: 10.1007/s41064-024-00329-4 30311153

[B25] Masse A. Ducrot D. Marthon P. (2012). “ Evaluation of supervised classification by class and classification characteristics”, in: Algorithms and technologies for multispectral, hyperspectral, and ultraspectral imagery XVIII, The proceedings are part of the series Proceedings of SPIE - The International Society for Optical Engineering (Maryland, United States: SPIE) 8390, 83902R. doi: 10.1117/12.919163

[B26] Mohanty S. Pandey P. C. Singh P. Dugesar V. Srivastava P. K. (2024). Vegetation discrimination based on chlorophyll prediction in marshy wetland using unmanned aerial vehicles. Aquat. Conserv. Mar. Freshw. Ecosyst. 34, e4170. doi: 10.1002/aqc.4170 42587375

[B27] Poursanidis D. Traganos D. Reinartz P. Chrysoulakis N. (2019). On the use of Sentinel-2 for coastal habitat mapping and satellite-derived bathymetry estimation using downscaled coastal aerosol band. Int. J. Appl. Earth Obs. Geoinf. 80, 58–70. doi: 10.1016/j.jag.2019.03.012 42574925

[B28] Priebe S. Huxley P. Knight S. Evans S. J. I. J. O. S. P. (1999). Application and results of the manchester short assessment of quality of life (MANSA). Int. J. Soc Psychiatry 45, 7–12. doi: 10.1177/002076409904500102 10443245

[B29] Probst P. Wright M. N. Boulesteix A.-L. (2019). Hyperparameters and tuning strategies for random forest. Wiley Interdiscip. Rev. Data Min. Knowl. Discov. 9, e1301. doi: 10.48550/arXiv.1804.03515

[B30] Qin H. Luo J. Xu Y. Xin Y. Xiao Q. Qiu Y. . (2025). Estimation models for pixel-scale coverage of aquatic vegetation in lakes based on Landsat and Sentinel data. J. Remote Sens. 5, 616. doi: 10.34133/remotesensing.0616

[B31] Qu G. Shuai Y. Huang Y. Ma X. Huo S. Madal A. P. (2026). Retrieval of fine resolution land covers in high latitude region of Alaska from integrated phenological characteristics. Sci. Remote Sens. 13, 100432. doi: 10.1016/j.srs.2026.100432 42574925

[B32] Raza D. Shu H. Aslam H. Nazeer M. Fan H. Nichol J. E. . (2026). Development of a spectral library (CROPSPECPK) for major crops through spectroscopy modeling and in-situ spectrometer data. BMC Plant Biol. 26, 561. doi: 10.1186/s12870-026-08387-z 41721250 PMC13032396

[B33] Rei S. Yuki Y. Hiroshi T. Xiufeng W. Nobuyuki K. Kan-ichiro M. (2018). Crop classification from Sentinel-2-derived vegetation indices using ensemble learning. J. Appl. Remote Sens. 12, 26019. doi: 10.1117/1.JRS.12.026019 42292344

[B34] Roujean J.-L. Breon F.-M. (1995). Estimating PAR absorbed by vegetation from bidirectional reflectance measurements. Remote Sens. Environ. 51, 375–384. doi: 10.1016/0034-4257(94)00114-3

[B35] Rouse J. W. Jr Haas R. H. Deering D. Schell J. Harlan J. C. (1974). “ Monitoring the Vernal Advancement and Retrogradation (Green Wave Effect) of Natural Vegetation⤷. Type II Report, NASA-CR-144661, RSC-1978-4, E75-10354. (College Station, TX: Texas A&M University Remote Sensing Center. Prepared for Goddard Space Flight Center under Contract NAS5-21857).

[B36] Rusňák T. Kasanický T. Malík P. Mojžiš J. Zelenka J. Sviček M. . (2023). “ Crop mapping without labels: Investigating temporal and spatial transferability of crop classification models using a 5-year Sentinel-2 series and machine learning,” in Remote Sens, vol. 15. (Basel: MDPI), 3414. doi: 10.3390/rs15133414

[B37] Shen Q. He D. Liu X. Shi Q. (2025). Integrating phenology knowledge graph into parcel-scale crop classification using multi-period deep time series modelling. Int. J. Appl. Earth Obs. Geoinf. 143, 104809. doi: 10.1016/j.jag.2025.104809 42574925

[B38] Sheng H. Zheng H.-X. (2023). Wetland information extraction based on multifeature optimization of multitemporal Sentinel-2 images. Mar. Sci. 47, 102–112.

[B39] Shukla G. Garg R. D. Srivastava H. S. Garg P. K. J. J. O. A. R. S. (2017). Implementation of random forest algorithm for crop mapping across an aridic to ustic area of Indian states. J. Appl. Remote Sens. 11, 026005–026005. doi: 10.1117/1.JRS.11.026005 42292344

[B40] Tiwari A. Zhao L. Sarkar S. Urutyan V. Vysyaraju U. S. R. Moon S. . (2026). Transferability of spatial and temporal learning models for winter wheat mapping in data-scarce environments: A case study in Armenia. Remote Sens. Appl. Soc Environ. 41, 101885. doi: 10.1016/j.rsase.2026.101885 42574925

[B41] Tufail R. Tassinari P. Torreggiani D. (2025). Assessing feature extraction, selection, and classification combinations for crop mapping using Sentinel-2 time series: A case study in northern Italy. Remote Sens. Applications: Soc. Environ. 38, 101525. doi: 10.1016/j.rsase.2025.101525 42574925

[B42] Van Deventer H. Linström A. Naidoo L. Job N. Sieben E. J. J. Cho M. A. (2022). Comparison between Sentinel-2 and WorldView-3 sensors in mapping wetland vegetation communities of the Grassland Biome of South Africa, for monitoring under climate change. Remote Sens. Appl. Soc Environ. 28, 100875. doi: 10.1016/j.rsase.2022.100875 42574925

[B43] Villa P. Berton A. Bolpagni R. Caccia M. Castellani M. B. Dalla Vecchia A. . (2025). Exploring spectral and phylogenetic diversity links with functional structure of aquatic plant communities. Remote Sens. Environ. 318, 114582. doi: 10.1016/j.rse.2024.114582 42574925

[B44] Wang J. (2015). Remote Sensing Monitoring and Environmental Management of Environmental Changes in the Poyang Lake Area (Beijing: Science Press).

[B45] Wang C. Chen J. Cai C. Liu M. Wang Z. Zhang H. . (2025). Comprehensive analysis of a parcel-level crop mapping (PARCM) framework from the perspective of spatial heterogeneity and spatiotemporal transferability. IEEE J. Sel. Top. Appl. Earth Obs. Remote Sens. 18, 7290–7303. doi: 10.1109/jstars.2025.3532283 25079929

[B46] Wang C. Chi J. Zhang X. Zhang N. (2026). Deep learning-based phenology extraction and crop classification in arid oasis using Sentinel-2 time series. J. Zhejiang University-SCIENCE B. 27, 537–560. doi: 10.1631/jzus.B2500403 42152569 PMC13183482

[B47] Wang L. Dronova I. Gong P. Yang W. Li Y. Liu Q. (2012). A new time series vegetation–water index of phenological–hydrological trait across species and functional types for Poyang Lake wetland ecosystem. Remote Sens. Environ. 125, 49–63. doi: 10.1016/j.rse.2012.07.003 42574925

[B48] Wei S. Zhang H. Wang C. Wang Y. Xu L. (2019). Multi-temporal SAR data large-scale crop mapping based on U-Net model. Remote Sens. 11, 68. doi: 10.3390/rs11010068 30654563

[B49] Wijesingha J. Dzene I. Wachendorf M. (2024). Evaluating the spatial–temporal transferability of models for agricultural land cover mapping using Landsat archive. ISPRS J. Photogramm. Remote Sens. 213, 72–86. doi: 10.1016/j.isprsjprs.2024.05.020 42574925

[B50] Wolf P. Rößler S. Schneider T. Melzer A. (2017). Collecting in situ remote sensing reflectances of submersed macrophytes to build up a spectral library for lake monitoring. Eur. J. Remote Sens. 46, 401–416. doi: 10.5721/EuJRS20134623 20206719

[B51] Wu Q. Hao Z. Sun C. Yan H. Yang Z. Dai S. J. W. S. (2017). Study on remote sensing monitoring method of aquatic medicine materials Euryale Ferox Salisb based on multi-source satellite remote sensing image. World Sci. Technology-Modernization Traditional Chin. Med. 19, 1787–1793.

[B52] Xin Y. Luo J. Xu Y. Sun Z. Qi T. Shen M. . (2024). SSAVI-GMM: An automatic algorithm for mapping submerged aquatic vegetation in shallow lakes using Sentinel-1 SAR and Sentinel-2 MSI data. IEEE Trans. Geosci. Electron. 62, 1–10. doi: 10.1109/tgrs.2024.3489883 25079929

[B53] Xin Y. Luo J. Zhai J. Wang K. Xu Y. Qin H. . (2025). An automatic algorithm for mapping algal blooms and aquatic vegetation using Sentinel-1 SAR and Sentinel-2 MSI data. Land 14, 592. doi: 10.3390/land14030592 30654563

[B54] Xue B. Chen G. Zhou R. Li J. Xu J. Sun F. . (2026). Predicting the potential distribution of Euryale ferox in China under future climate scenarios using MaxEnt modeling. Sci. Rep. 16, e1301. doi: 10.1038/s41598-026-54261-1 42162212 PMC13392123

[B55] Yu X. Bian S. Zhou X. Wang G. He H. An W. (2014). “ A stepwise refinement classification method of remote sensing image based on feedback strategies”, in: 2014 IEEE Geoscience and Remote Sensing Symposium (Piscataway, NJ: IEEE (Institute of Electrical and Electronics Engineers)).

[B56] Zarco‐Tejada P. J. Ustin S. L. Whiting M. L. (2005). Temporal and spatial relationships between within‐field yield variability in cotton and high‐spatial hyperspectral remote sensing imagery. Agron. J. 97, 641–653. doi: 10.2134/agronj2003.0257

[B57] Zhang F. Liang S. Ma H. Li W. Chen Y. He T. . (2026). A review of crop yield estimation on pixel and field scales from remotely sensed data. Sci. Remote Sens. 13, 100342. doi: 10.1016/j.srs.2025.100342 42574925

