# Supplementary material for: Spatiotemporal transferability of multi-temporal random forest model for mapping Euryale ferox Salisb. plantations using Sentinel-2 imagery
Source: Front Plant Sci. 2026 Sep 3;17:1932872. doi: 10.3389/fpls.2026.1932872 (PMC13582415; doi:10.3389/fpls.2026.1932872)
Supplement: Supplementary file 1 [file DataSheet1.docx]

Supplementary Material

# 1 Supplementary Figures and Tables

## 1.1 Supplementary Figures

**Supplementary Figure 1.** Based on the established random forest models, the classification maps of Jinxian County in 2025 were generated. Panels (a–d) represent the output classification maps from the July, August, September, and multi-temporal RF models in Jinxian County in 2025, respectively; (a1–d1) and (a2–d2) are the corresponding detailed views of two specific areas for the four models, respectively.

**Supplementary Figure 2.** Multi-temporal RF model confusion matrix

## 1.2 Supplementary Tables

**Supplementary Table 2. Details of Sentinel-2 remote sensing data used in this study**

| **Region** | **Time** | **Cloud cover** | **Product Level** | **Data Usage** |
| --- | --- | --- | --- | --- |
| N0510_R132_T50RMT | 20240525 | 5.91% | S2A_L1C | Calibration |
| N0510_R132_T50RMS | 20240525 | 23.98% | S2A_L1C | Calibration |
| N0510_R132_T50RMT | 20240704 | 3.65% | S2A_L2A | Calibration |
| N0510_R132_T50RMS | 20240704 | 0.71% | S2A_L2A | Calibration |
| N0511_R132_T50RMT | 20240803 | 7.45% | S2A_L2A | Calibration |
| N0511_R132_T50RMS | 20240803 | 1.57% | S2A_L2A | Calibration |
| N0511_R132_T50RMT | 20240902 | 6.87% | S2A_L2A | Calibration |
| N0511_R132_T50RMS | 20240902 | 11.82% | S2A_L2A | Calibration |
| N0511_R132_T50RMT | 20241002 | 23.44% | S2A_L1C | Calibration |
| N0511_R132_T50RMS | 20241002 | 21.51% | S2A_L1C | Calibration |
| N0511_R132_T50RMT | 20250704 | 23.95% | S2B_L2A | Validation |
| N0511_R132_T50RMS | 20250704 | 15.13% | S2B_L2A | Validation |
| N0511_R132_T50RMT | 20250808 | 24.68% | S2C_L2A | Validation |
| N0511_R132_T50RMS | 20250808 | 2.86% | S2C_L2A | Validation |
| N0511_R132_T50RMT | 20250828 | 2.12% | S2C_L2A | Validation |
| N0511_R132_T50RMS | 20250828 | 0.18% | S2C_L2A | Validation |
